# A randomized, phase II study of sequential belimumab and rituximab in primary Sjögren’s syndrome

**DOI:** 10.1172/jci.insight.163030

**Published:** 2022-12-08

**Authors:** Xavier Mariette, Francesca Barone, Chiara Baldini, Hendrika Bootsma, Kenneth L. Clark, Salvatore De Vita, David H. Gardner, Robert B. Henderson, Michael Herdman, Karoline Lerang, Prafull Mistry, Raj Punwaney, Raphaele Seror, John Stone, Paul L.A. van Daele, André van Maurik, Nicolas Wisniacki, David A. Roth, Paul Peter Tak

**Affiliations:** 1 Department of Rheumatology, Université Paris-Saclay, Hôpital Bicêtre, Assistance Publique — Hôpitaux de Paris, INSERM UMR1184, Le Kremlin Bicêtre, Paris, France.; 2 Institute of Inflammation and Ageing, College of Medical and Dental Sciences, University of Birmingham, Birmingham, United Kingdom.; 3 Centro Farmacologia Clinica AOUP, Rheumatology Unit, Department of Clinical and Experimental Medicine, University of Pisa, Pisa, Italy.; 4 Department of Rheumatology and Clinical Immunology, University of Groningen, University Medical Center Groningen, Groningen, The Netherlands.; 5 Clinical Science, GSK, Stevenage, Hertfordshire, United Kingdom.; 6 Rheumatology Clinic, Department of Medical Area, Azienda Ospedaliera Universitaria di Udine, Udine, Italy.; 7 Clinical Pharmacology and Experimental Medicine, GSK, Stevenage, Hertfordshire, United Kingdom.; 8 Department of Rheumatology, Oslo University Hospital, Oslo, Norway.; 9 R&D Biostatistics, GSK, Stevenage, Hertfordshire, United Kingdom.; 10 Pharmaceutical Research and Development, GSK, Collegeville, Pennsylvania, USA.; 11 R&D, GSK, Stevenage, Hertfordshire, United Kingdom.; 12 Department of Internal Medicine and Department of Immunology, Erasmus Medical Center, Rotterdam, The Netherlands.; 13 Discovery Medicine, GSK, Stevenage, Hertfordshire, United Kingdom.; 14 R&D, GSK, Collegeville, Pennsylvania, USA.

**Keywords:** Clinical Trials, Immunology, Autoimmune diseases, Drug therapy

## Abstract

**BACKGROUND:**

Primary Sjögren’s syndrome (pSS) is characterized by B cell hyperactivity and elevated B-lymphocyte stimulator (BLyS). Anti-BLyS treatment (e.g., belimumab) increases peripheral memory B cells; decreases naive, activated, and plasma B cell subsets; and increases stringency on B cell selection during reconstitution. Anti-CD20 therapeutics (e.g., rituximab) bind and deplete CD20-expressing B cells in circulation but are less effective in depleting tissue-resident CD20^+^ B cells. Combined, these 2 mechanisms may achieve synergistic effects.

**METHODS:**

This 68-week, phase II, double-blind study (GSK study 201842) randomized 86 adult patients with active pSS to 1 of 4 arms: placebo, s.c. belimumab, i.v. rituximab, or sequential belimumab + rituximab.

**RESULTS:**

Overall, 60 patients completed treatment and follow-up until week 68. The incidence of adverse events (AEs) and drug-related AEs was similar across groups. Infections/infestations were the most common AEs, and no serious infections of special interest occurred. Near-complete depletion of minor salivary gland CD20^+^ B cells and a greater and more sustained depletion of peripheral CD19^+^ B cells were observed with belimumab + rituximab versus monotherapies. With belimumab + rituximab, reconstitution of peripheral B cells occurred, but it was delayed compared with rituximab. At week 68, mean (± standard error) total EULAR Sjögren’s syndrome disease activity index scores decreased from 11.0 (1.17) at baseline to 5.0 (1.27) for belimumab + rituximab and 10.4 (1.36) to 8.6 (1.57) for placebo.

**CONCLUSION:**

The safety profile of belimumab + rituximab in pSS was consistent with the monotherapies. Belimumab + rituximab induced enhanced salivary gland B cell depletion relative to the monotherapies, potentially leading to improved clinical outcomes.

**TRIAL REGISTRATION:**

ClinicalTrials.gov NCT02631538.

**FUNDING:**

Funding was provided by GSK.

## Graphical abstract

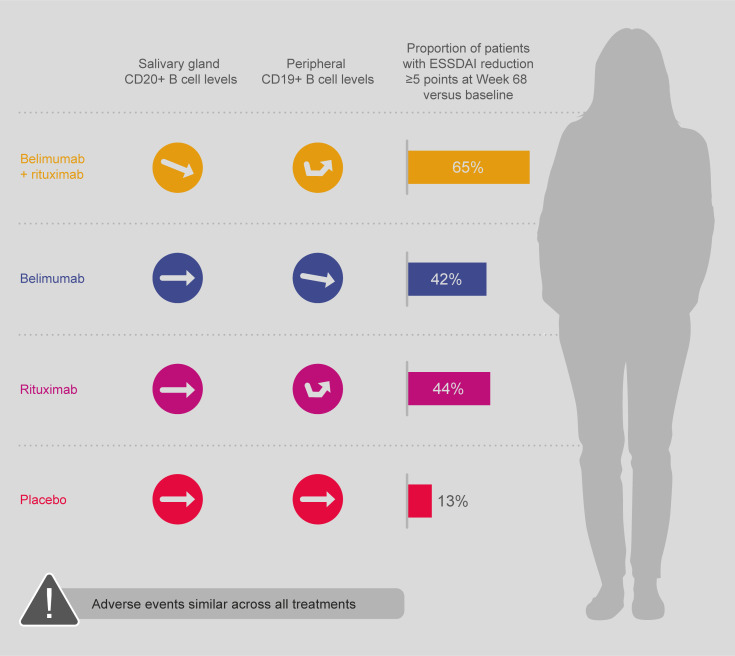

## Introduction

Primary Sjögren’s syndrome (pSS) is an autoimmune disease characterized by a wide clinical presentation spectrum including oral and ocular dryness, constitutional symptoms, and potentially severe organ-specific extraglandular manifestations (1–3). Abundance of focal B cell infiltrates and formation of ectopic germinal centers (GCs) in the salivary glands are characteristic of pSS and correlate with systemic manifestations (3, 4). Additionally, elevated levels of B-lymphocyte stimulator (BLyS, also known as B cell activating factor [BAFF], which promotes B cell maturation, proliferation, and survival) are found in the serum and saliva of patients with pSS, with serum BLyS levels correlating with markers of disease activity, including salivary gland B cell clonal expansion, lymphoproliferation, and pSS-associated autoantibodies (anti–Ro/SS-A, anti–La/SS-B), IgG, and rheumatoid factor (RF) (5–8). Furthermore, high levels of chemokine (C-X-C motif) ligand 13 (CXCL13) — which plays a key role in the maintenance of ectopic tertiary lymphoid structures, organization of B cell follicles, and migration of B cells into ectopic GCs — have been associated with high pSS disease activity (9–13). There are no approved disease-modifying treatments for pSS (14).

Belimumab is an anti-BLyS mAb treatment approved for active systemic lupus erythematosus (SLE) and active lupus nephritis (LN) (15–20). In pSS, treatment with belimumab has been associated with symptom improvement in up to 60% of patients in the open-label, phase II BELISS study, measured by significant decreases in both the European League Against Rheumatism (EULAR) Sjögren’s Syndrome Disease Activity Index (ESSDAI) and the EULAR Sjögren’s Syndrome Patient Reported Index (ESSPRI) (21). As a result, EULAR recommends that belimumab be considered as a rescue therapy in cases of severe, refractory, systemic pSS (22). Ianalumab, a human IgG1 mAb that targets the BAFF receptor, yielded promising results in a preliminary phase II study (23). In a further phase II study, ianalumab showed significant improvements in ESSDAI, Physicians Global Assessment, and stimulated salivary flow versus placebo (24).

Rituximab, used for the treatment of several autoimmune diseases and B cell hematologic malignancy (25, 26), is an anti-CD20 mAb that binds to and depletes CD20-expressing B cells (27, 28). Studies of rituximab treatment in pSS have shown varied results (25, 26, 29–35). Two large placebo-controlled trials (TEARS and TRACTISS) failed to meet their primary efficacy end point (34, 36); however, response rates were significantly greater with rituximab versus placebo in a post hoc analysis that reassessed TRACTISS data using the Composite of Relevant Endpoints for Sjögren’s Syndrome (CRESS) composite end point (37). Rituximab is not approved for the treatment of pSS; however, according to EULAR recommendations, rituximab may be considered in severe, refractory, systemic pSS (22, 38).

The limited efficacy of rituximab in pSS might be explained by the finding that the administration of rituximab leads to increased levels of serum BLyS that, in turn, favor the reemergence of autoreactive B cells during B cell repopulation, leading to disease relapse over time (39–42). Additionally, although peripheral B cells are quickly depleted by rituximab, tissue-resident CD20^+^ B cells in microenvironments such as mucosa-associated lymphoid tissue (MALT) and inflamed tissues are less responsive to the depleting effect of rituximab (27, 43–45). The resistance of CD20^+^ B cells to rituximab-induced B cell depletion in the tissues is attributed, at least in part, to the ectopic expression of BLyS in inflamed sites that may compromise rituximab-induced lysis by NK cells, as well as enhance the survival of autoreactive B cells (46–48). For this reason, sequential treatment with belimumab and rituximab (belimumab + rituximab) could provide a strategy for treating autoantibody-positive autoimmune diseases, as 2 complementary mechanisms would be combined (39, 49–51). In contrast to the resistance seen in tissue to rituximab-mediated B cell depletion, belimumab decreases naive, activated, and plasma B cell subsets but spares memory B cells, as evidenced by a rapid increase in peripheral memory B cells following BLyS neutralization. This is possibly due to the disruption of memory B cell trafficking, either by mobilization of memory B cells into the circulation or by blocking memory B cells accessing the tissues (17, 51, 52). Anti-CD20 therapeutics, such as rituximab, eliminate peripheral B cells (including CD20^+^ memory B cells) through complement-dependent cytotoxicity and antibody-dependent, cell-mediated cytotoxicity (27, 53, 54). Belimumab-induced increase in circulating memory B cells and/or direct in situ effects by belimumab are likely to render B cells more susceptible to rituximab-mediated depletion (39, 51). Lastly, continued BLyS neutralization following a single cycle of rituximab is hypothesized to increase the stringency on B cell selection during reconstitution, thus compromising the survival of autoreactive B cells, in particular (39). The usefulness of sequential or combined treatment of belimumab and rituximab is supported by case reports showing improvement with belimumab either preceding rituximab in pSS (50, 51) or following rituximab in pSS, SLE, and LN (55–57).

As the safety profile of the combination has yet to be established, we conducted a 68-week study and evaluated the effects of s.c. belimumab administered with a single cycle of rituximab on safety, biomarkers, and clinical efficacy compared with placebo and monotherapies in adult patients with pSS.

## Results

### Patients

**Figure 1:**
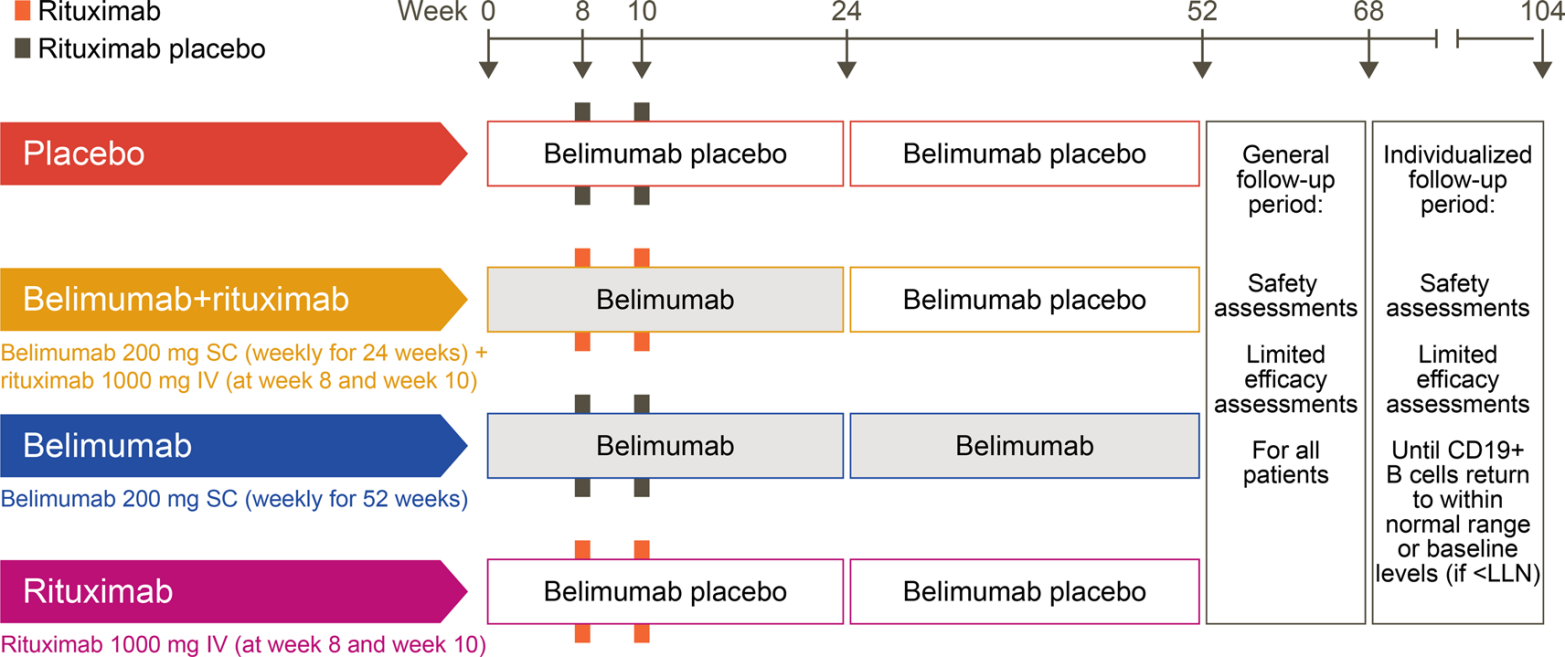
Study design. LLN, lower limit of normal.

**Figure 2:**
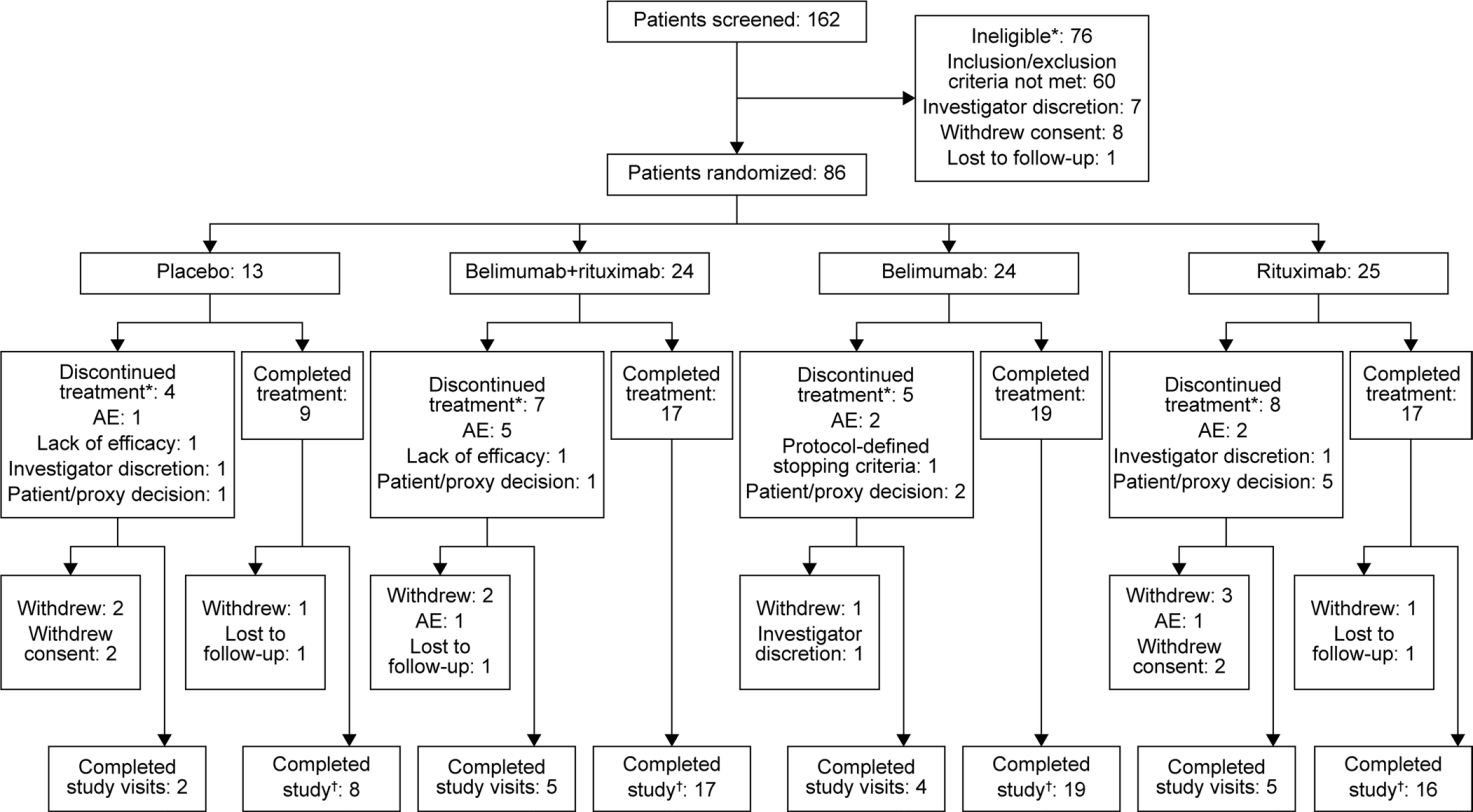
Patient flow through the study. *Patients may only have one primary reason;.†Completer population (patients who completed the 52-week treatment and general follow-up period of the study, including the visit at week 68). AE, adverse event.

The first patient was enrolled on February 17, 2016, and the last patient completed the study on June 23, 2020 (study design shown in Figure 1). A total of 86 patients received ≥ 1 dose of their intended study treatment (safety population, Figure 2). Most enrolled patients (72.1% [*n* = 62/86]) completed the 52-week study treatment period, and 69.8% (*n* = 60/86) also completed the 16-week follow-up period (completer population). The proportion of completers was slightly higher in active treatment groups versus placebo (70.8% [*n* = 17/24] belimumab + rituximab, 79.2% [*n* = 19/24] belimumab, 64.0% [*n* = 16/25] rituximab, versus 61.5% [*n* = 8/13] placebo). Of patients in the safety population, 18.6% (*n* = 16/86) withdrew from treatment but continued study visits, and 11.6% (*n* = 10/86) withdrew from the study. The most common reasons for withdrawal were withdrawal of consent (4.7% [*n* = 4/86]) and loss to follow-up (3.5% [*n* = 3/86]). Of the 60 patients who completed the week-68 visit, 73.3% (*n* = 44/60) entered the individualized safety off-treatment follow-up period.

Baseline demographics and disease characteristics were generally similar across treatment groups (Table 1 and Table 2, respectively). Most patients were female (91.7%–100.0%) and White (83.3%–92.3%; Table 1). Patients had moderate-to-severe baseline disease activity, with a mean ESSDAI (± SD) score range across groups of 10.3–12.2 (5.98–5.23). The median (minimum, maximum) ESSDAI score range across groups was 7.5 (5, 31) to 12.0 (6, 25); the lowest and highest overall ESSDAI scores were 5 and 31.

Of the safety population, 50.0% (*n* = 12/24) of the patients in the belimumab + rituximab group, 75.0% (*n* = 18/24) of the patients in the belimumab group, 48.0% (*n* = 12/25) of the patients in the rituximab group, and 15.4% (*n* = 2/13) of the patients in the placebo group continued to have B cell levels below the lower limit of normal at the end of the study treatment period and were required, per protocol, to enter an individualized safety follow-up period of up to 36 weeks.

### Safety outcomes

#### Exposure.

Treatment compliance was high and treatment duration was similar across treatment groups (median, 364 days in all groups). The planned number of belimumab/placebo injections was 52; the median total number of injections received was 52 in the belimumab group and 51 in the other groups. Most patients (87.5% [*n* = 21/24] belimumab + rituximab and belimumab, 80.0% [*n* = 20/25] rituximab, 92.3% [*n* = 12/13] placebo) received both week 8 and week 10 rituximab/placebo infusions.

#### Adverse events.

The proportion of patients experiencing ≥ 1 adverse event (AE) and the incidence of drug-related AEs were similar across treatment groups (Table 3). The most common AEs by system organ class were infections and infestations, musculoskeletal and connective tissue disorders, and general disorders and administration site conditions. The high incidence of infections and infestations was primarily driven by nasopharyngitis, while the high incidence of musculoskeletal and connective tissue disorders was primary driven by arthralgia. None of the patients developed additional connective tissue disorders (other than pSS) during the study period. The proportions of patients with AEs in the belimumab + rituximab, belimumab, and rituximab groups were similar to or lower than the proportion of patients with AEs in the placebo group for most system organ classes (Table 3). Psychiatric disorders represented the only system organ class with a > 20% higher proportion of patients experiencing AEs in any active treatment group when compared with patients in the placebo group. In addition, 2 AE cases were reported with the preferred term serum sickness: 1 in the belimumab + rituximab group and 1 in the rituximab group. The most common (incidence ≥ 5% in any treatment group) grade 2–4 AEs (moderate to potentially life-threatening) were arthralgia and pneumonia. Most AEs were mild or moderate in severity.

A higher proportion of patients experienced AEs resulting in study drug discontinuation in the active treatment groups (*n* = 5/24 [20.8%] belimumab + rituximab, *n* = 3/24 [12.5%] belimumab, *n* = 5/25 [20.0%] rituximab) compared with placebo (*n* = 1/13 [7.7%]; Figure 2). A review of these AEs in the belimumab + rituximab and rituximab groups revealed that they were disparate and showed no apparent relationship to the timing of the rituximab infusion.

Serious AEs (SAEs) did not occur in the placebo group; however, SAEs did occur in 3 (12.5%) patients in the belimumab + rituximab group, 2 (8.3%) patients in the belimumab group, and 4 (16.0%) patients in the rituximab group (Table 3). No specific SAEs were experienced by > 1 patient. SAEs considered at least possibly related to study drug were reported in 2 (8.3%) patients in the belimumab + rituximab group (enterocolitis infectious and pyelonephritis), 1 (4.2%) patient in the belimumab group (pneumonia), and 1 (4.0%) patient in the rituximab group (neutropenia and rash). Infection and infestation SAEs were reported in 2 (8.3%) patients in the belimumab + rituximab group, 1 (4.2%) patient in the belimumab group, and 1 (4.0%) patient in the rituximab group (Supplemental Table 1; supplemental material available online with this article; https://doi.org/10.1172/jci.insight.163030DS1). One death was reported in the belimumab + rituximab group (food aspiration), considered by the investigator to be unrelated to the study drug (Table 3).

#### Adverse events of special interests (AESIs).

No malignant neoplasms were reported except for nonmelanoma skin cancer, of which there were 2 events reported for 1 patient in the rituximab group (Table 4). There were no imbalances in postadministration systemic reactions (PASR) or infections of special interest (opportunistic infections, herpes zoster, tuberculosis, sepsis) between active treatment and placebo. There were no cases of serious infections of special interest. Incidence of depression (including mood disorders and anxiety) was higher in all active treatment groups (*n* = 3/24 [12.5%] belimumab + rituximab, *n* = 5/24 [20.8%] belimumab, *n* = 1/25 [4.0%] rituximab) compared with placebo (*n* = 0/13 [0.0%]); 1 patient in the rituximab group experienced a suicide/self-injury event (ideation) of moderate severity and resulted in discontinuation of treatment. The patient subsequently recovered and continued study visits.

#### Study-specific AESI.

There were no reports of severe skin reactions, posterior reversible encephalopathy syndrome, or progressive multifocal leukoencephalopathy during the study. Cardiac disorders were reported for 1 (4.2%) patient in the belimumab + rituximab group (atrial flutter) and 1 (4.0%) patient in the rituximab group (acute cardiac failure); both were serious, and 1 (acute cardiac failure) led to discontinuation of study treatment. The atrial flutter occurred 230 days after the second rituximab infusion, and the cardiac failure occurred 38 days after study treatment started (i.e., before rituximab infusion).

#### AEs during the individualized safety follow-up period.

In total, 54 AEs were reported in 22 patients during the individualized follow-up period, and there were 2 SAEs (aortic valve stenosis and cerebrovascular accident) that were unrelated to the study drug and were resolved. The most commonly reported AEs during the individualized follow-up period fell within the infections and infestations system organ class (18 events in 15 patients) and gastrointestinal disorders system organ class (17 events in 4 patients). Overall, there were no safety concerns and no additional AESI identified from the events reported during the individualized follow-up period.

### Immunological outcomes

#### B cells in the peripheral blood.

**Figure 3:**
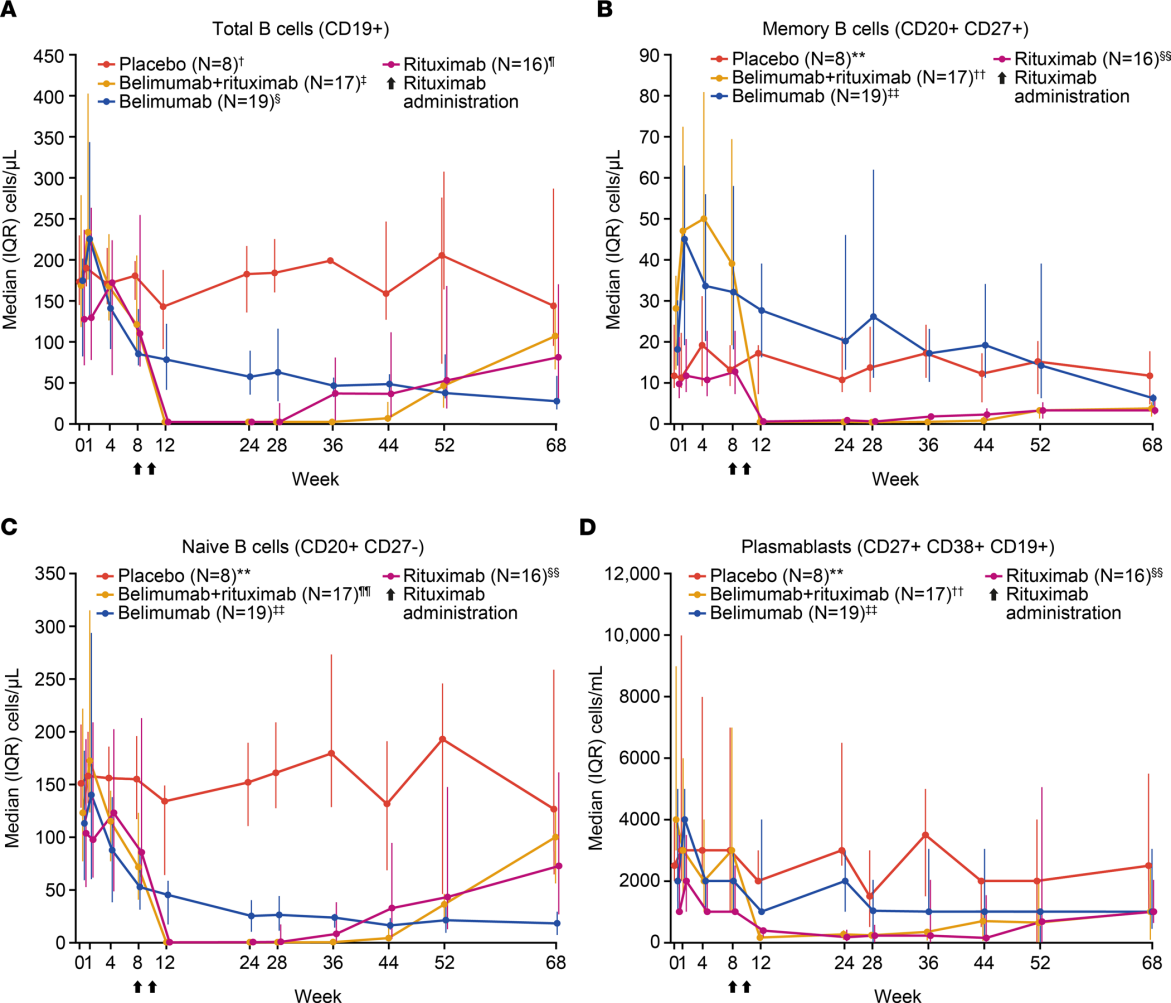
Median (IQR). (**A**–**D**) Total B cells (CD19^+^), memory B cells (CD20^+^CD27^+^), naive B cells (CD20^+^CD27^–^), and plasmablasts (CD27^+^CD38^+^CD19^+^) over time by flow cytometry (completer population, *n* = 60). Flow cytometry data were analyzed using the Hodges-Lehmann method to provide a nonparametric 95% CI for the treatment comparisons of interest. For clear presentation of results, data in **A**–**C** are presented as cells/μL (with different *y* axes maximum values), and data in **D** are presented as cells/mL. ^†^*N* = 7 at weeks 4, 12, and 52. ^‡^*N* = 16 at weeks 1, 36, 44, 68. ^§^*N* = 17 at weeks 1, 44, and 68. *N* = 18 at weeks 4, 12, 28, and 36. *N* = 16 at week 52. ^¶^*N* = 13 at week 24. **N = 7 at weeks 4, 8, and 12. *N* = 6 at week 52. ^††^*N* = 16 at weeks 1, 8, 44, and 68. *N* = 15 at week 36. ^‡‡^*N* = 17 at weeks 1, 44, and 68. *N* = 18 at weeks 4, 8, 28, and 36. *N* = 16 at week 12. *N* = 15 at week 52. ^§§^*N* = 13 at week 24. *N* = 14 at week 36. ^¶¶^*N* = 16 at weeks 1, 8, 28, 44, and 68. *N* = 15 at weeks 24 and 36. IQR, interquartile range.

As shown by flow cytometry, belimumab treatment led to early reductions in the number of peripheral total B cells (CD19^+^) and B cell subsets, including naive B cells (CD20^+^CD27^–^) and plasmablasts (CD27bright^+^CD38bright^+^CD19^+^ [CD27br^+^CD38br^+^CD19^+^]), whereas peripheral memory B cells (CD20^+^CD27^+^) initially increased (Figure 3). Belimumab-induced increase in peripheral memory B cells was observed in both switched (CD19^+^CD27^+^IgD^–^) and nonswitched memory B cells (CD19^+^CD27^+^IgD^+^) (Supplemental Figure 1). Peripheral memory B cells gradually decreased to below baseline levels in patients treated with belimumab, while patients treated with rituximab experienced an immediate and marked reduction in peripheral memory B cells, as well as in other CD20^+^ B cell subsets (Figure 3).

Total peripheral blood B cells, as well as other B cell subsets in the circulation, were almost completely depleted (reaching the lower limit of quantification [2.5 cells/μL]) in the belimumab + rituximab and rituximab groups (Figure 3A). There was a trend toward delayed repopulation of total B cells in the circulation in the belimumab + rituximab group after belimumab was discontinued at week 24 (Figure 3A). Repopulation of total B cells in the rituximab group was apparent at week 36; however, median levels of total B cells in the belimumab + rituximab group did not reach a similar level until week 52. In contrast, memory B cells in the belimumab + rituximab group remained suppressed to week 68, with no apparent differences between the belimumab + rituximab and rituximab groups after week 12 (Figure 3B). There were no notable changes or treatment differences in CD3^+^CD4^+^ and CD3^+^CD8^+^ T cell counts for any of the active treatment groups compared with placebo throughout the study (Supplemental Figure 2).

#### Biomarkers in the peripheral blood.

**Figure 4:**
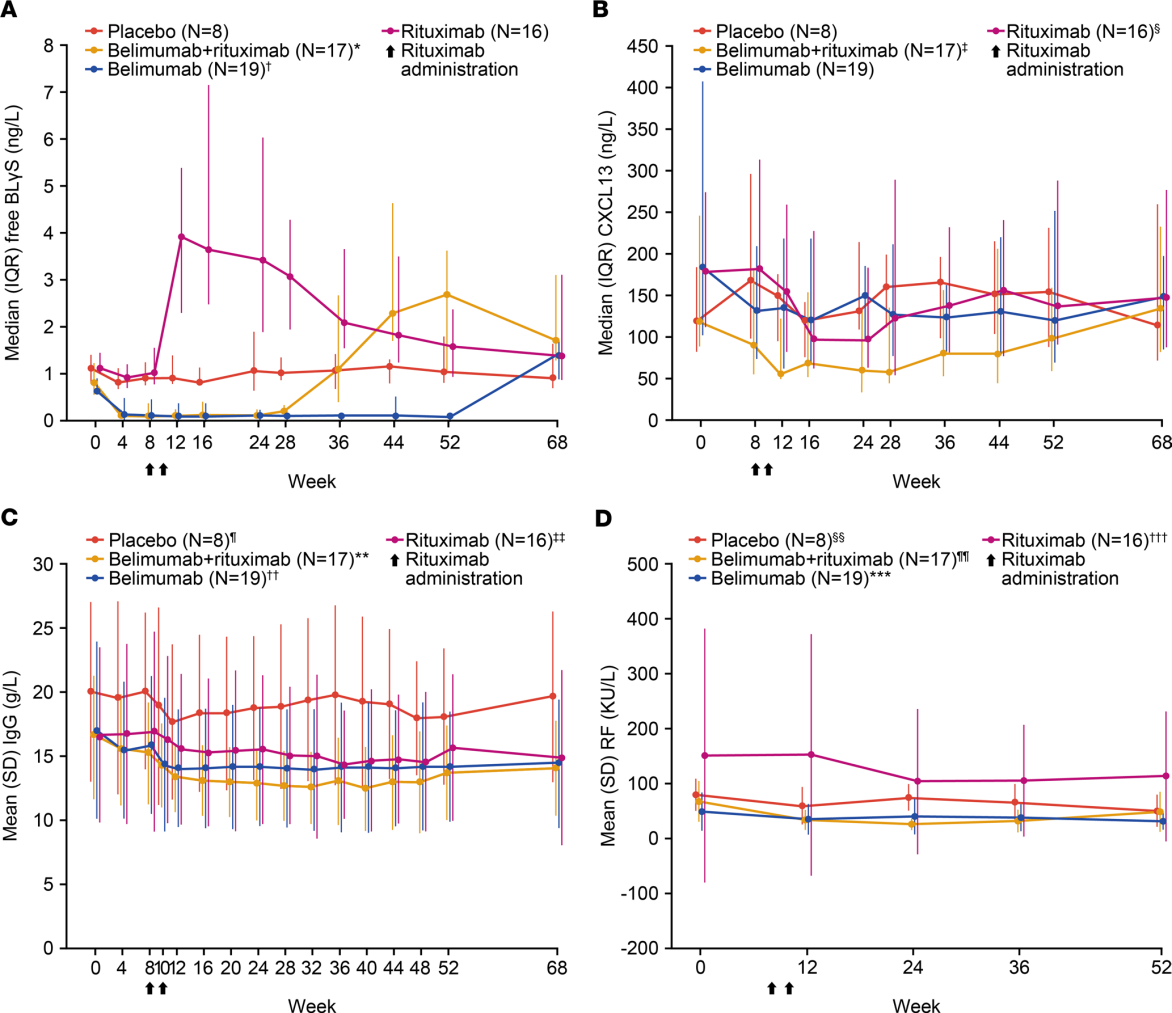
Key serological biomarkers over time. (**A**–**C**) Free BLyS, CXCL13, IgG, and RF (patients positive at baseline) (completer population, *n* = 60). *N = 16 at week 36. ^†^*N* = 18 at week 28. ^‡^*N* = 16 at weeks 36 and 44. ^§^*N* = 15 at week 24. ^¶^*N* = 7 at weeks 4 and 48. **N = 16 at week 8. ^††^*N* = 17 at week 20. *N* = 18 at week 36. ^‡‡^*N* = 15 at weeks 10, 24, 40, and 48. *N* = 14 at week 32. ^§§^*N* = 6 at weeks 0, 36, and 52. *N* = 7 at week 12. *N* = 5 at week 24. ^¶¶^*N* = 7 at week 0. *N* = 6 at week 12. *N* = 5 at weeks 24, 36, and 52. ***N = 14 at week 0. *N* = 12 at week 12. *N* = 10 at weeks 24 and 36. *N* = 9 at week 52. ^†††^*N* = 11 at week 0. *N* = 9 at weeks 12, 24, and 36. *N* = 10 at week 52. BLyS, B-lymphocyte stimulator; CXCL13, chemokine (C-X-C motif) ligand 13; IgG, immunoglobulin G; RF, rheumatoid factor.

Target engagement in the serum, as measured by total BLyS levels (free BLyS and BLyS complexed with belimumab), peaked at week 36 in the belimumab + rituximab and belimumab groups (Supplemental Table 2). Total BLyS levels remained elevated for 28–44 weeks after the last belimumab administration, with levels decreasing upon pharmacokinetic clearance (data not shown) and B cell repopulation (Supplemental Table 2). As expected, free BLyS levels increased immediately after B cell depletion by rituximab at week 12, whereas in the belimumab + rituximab group, the increase in free BLyS did not occur after treatment with rituximab but was observed 12 weeks after discontinuation of belimumab (Figure 4A).

Serum CXCL13 concentrations were measured as a possible surrogate biomarker of immunological activity in the salivary gland. A reduction in serum CXCL13 concentrations was observed in all 3 active treatment groups, with the most sustained effect observed in the belimumab + rituximab group (Figure 4B and Supplemental Table 2). Trends toward a reduction in IgA, IgM, and IgG, RF, serum κ and λ light chain levels were observed in the belimumab + rituximab group compared with placebo, but there was no clear differentiation between belimumab and rituximab groups (Figure 4 and Supplemental Table 2). There was no evidence for treatment-related normalization in SS-A, SS-B autoantibody titers, or β2-microglobulin, complement components 3 (C3) or C4, or hemolytic complement (CH50) levels (Supplemental Table 2).

#### B cells and biomarkers in the minor salivary gland.

**Figure 5:**
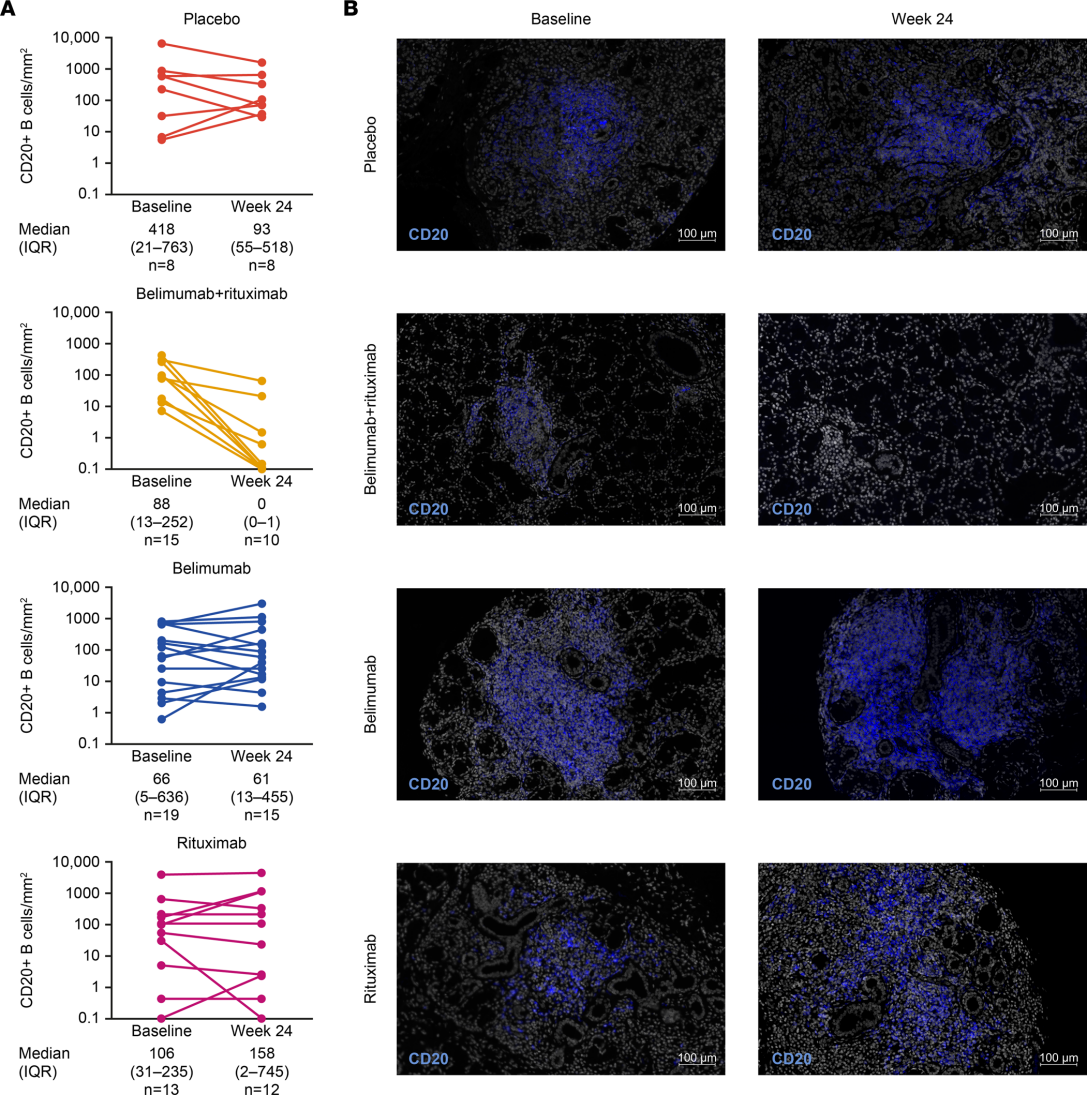
Absolute CD20^+^ B cells in the salivary gland and representative immunofluorescence images. (**A** and **B**) Absolute CD20^+^ B cells in the salivary gland and representative immunofluorescence (Hoechst CD20) histological images. (completer population, *n* = 60). Median (IQR): includes all baseline/week 24 completer data. Only data for patients with paired baseline/week 24 biopsies. Minimum values = 0.1. When CD20^+^ B cells were undetectable, values were input as 0.1 to allow logarithmic display. Changes in absolute CD20^+^ B cells in the salivary gland were analyzed using the Hodges-Lehmann method to provide a 95% CI for treatment comparisons of interest. For the histological images, original slides were imaged at 20× using a Zeiss Axio Scan Z1 slide scanner and are included in Supplemental Figure 3. IQR, interquartile range. Scale bars: 100 µm.

Minor salivary gland (MSG) histology showed a uniform reduction of the CD20^+^ B cell count at week 24 in the belimumab + rituximab group, with incomplete depletion prevailing in the belimumab, rituximab, and placebo groups (Figure 5 and Supplemental Figure 3). Levels of MSG-resident memory B cells (CD20^+^CD27^+^) were lowest in the belimumab + rituximab and rituximab groups but were spared in the belimumab group (Supplemental Table 3). Memory B cells were similarly depleted at week 24 for the belimumab + rituximab and rituximab monotherapy groups. Plasma cell (CD138^+^; both CD20^+^ and CD20^–^) numbers were spared irrespective of treatment, except for a slight reduction in the rituximab group.

The lowest lymphocyte focus scores (LFS) at week 24 were observed in the belimumab + rituximab and rituximab groups; however, scores failed to show a substantial difference in posttreatment samples (Supplemental Table 3). The greatest decrease in ratio of total aggregate area/total glandular area was also observed in the belimumab + rituximab group at week 24 compared with the belimumab, rituximab, and placebo groups.

### Clinical outcomes

**Figure 6:**
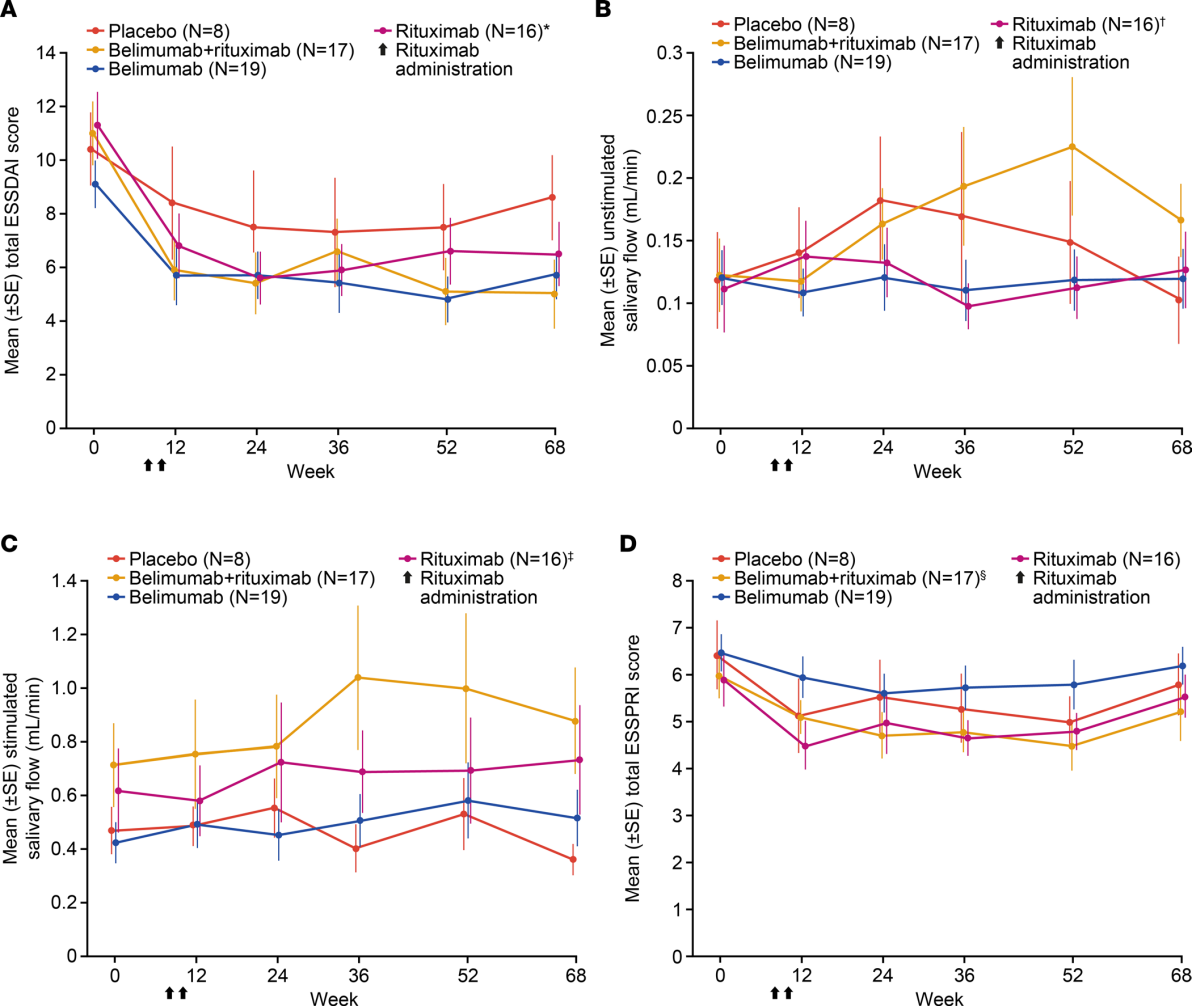
Clinical efficacy over time as measured by mean (standard error). (**A**–**D**) ESSDAI total score, unstimulated salivary flow, stimulated salivary flow, and ESSPRI total score (completer population, *n* = 60). *N = 15 at week 12. ^†^*N* = 15 at week 36. ^‡^*N* = 15 at weeks 36 and 68. ^§^*N* = 16 at week 52. ESSDAI, EULAR Sjögren’s syndrome disease activity index; ESSPRI, EULAR Sjögren’s Syndrome Patient Reported Index.

**Figure 7:**
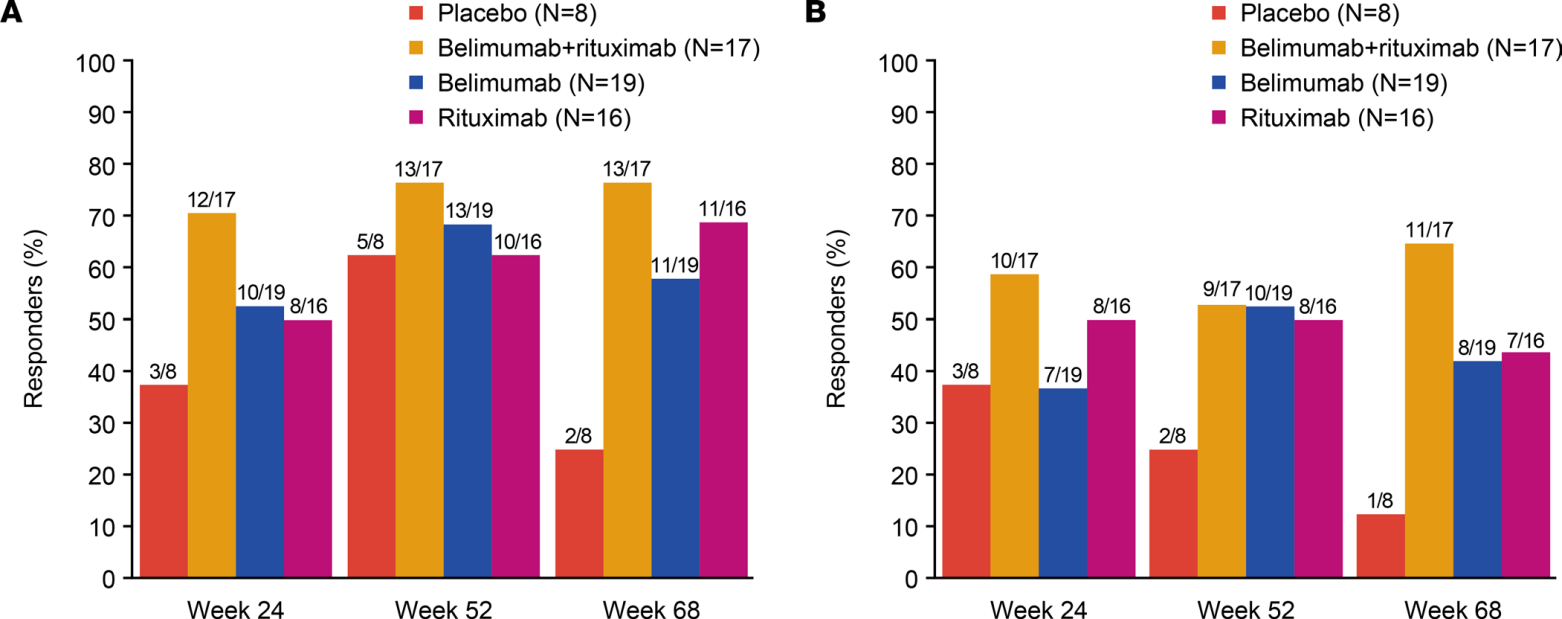
Proportion of responders with an ESSDAI reduction. (**A** and **B**) Proportion of responders with an ESSDAI reduction of ≥3 points and ≥5 points versus baseline (completer population, *n* = 60). ESSDAI responder analyses utilized a generalized estimating equation model. ESSDAI, EULAR Sjögren’s syndrome disease activity index.

There was a trend toward greater reduction in mean total ESSDAI score in the belimumab + rituximab group compared with placebo (Figure 6A). This was observed as early as week 12 and was sustained to week 68, which was 44 weeks after cessation of active treatment. A similar trend for improvement in total ESSDAI score was observed in the belimumab and rituximab groups compared with placebo. Mean (± standard error) total ESSDAI score was lower with belimumab + rituximab (5.0 [1.27]) at week 68 compared with either belimumab (5.7 [0.88]), rituximab (6.5 [1.18]), or placebo (8.6 [1.57]). The least–squares mean (± standard error) change from baseline in ESSDAI total score was greater in the belimumab + rituximab group at weeks 24 (–5.3 [0.91]), 52 (–5.7 [0.89]), and 68 (–5.7 [0.96]), compared with belimumab (week 24, –3.9 [0.87]; week 52, –4.8 [0.85]; week 68, –3.9 [0.92]), rituximab (week 24, –5.3 [0.94]; week 52, –4.3 [0.92]; week 68, –4.4 [0.99]), and placebo (week 24, –2.9 [1.32]; week 52, –2.9 [1.29]; week 68, –1.8 [1.40]) groups. Responder analysis for ESSDAI indicated that, compared with placebo, there was a numerically higher proportion of responders with belimumab + rituximab at weeks 24 and 52, which was sustained to week 68, where the difference versus other treatment arms was greatest (Figure 7). Furthermore, responder analysis for the Clinical ESSDAI (ClinESSDAI) indicated a numerically higher proportion of responders with belimumab + rituximab at week 24 and belimumab at week 36 compared with both placebo and rituximab, which was sustained to week 68 (Supplemental Table 4).

Accordingly, mean unstimulated salivary flow was greater with belimumab + rituximab versus placebo at weeks 52 and 68 (Figure 6B). A trend toward a higher stimulated salivary flow was also observed at weeks 52 and 68 with belimumab + rituximab compared with placebo, belimumab, and rituximab groups (Figure 6C). In contrast, there were no notable differences in patients reporting oral dryness with any treatment relative to placebo (Supplemental Figure 4). Compared with placebo, there were no notable differences with active treatment in mean total ESSPRI score (Figure 6D), ESSPRI domain scores (Supplemental Figure 5), or lacrimal gland function (Supplemental Table 4).

## Discussion

This phase II clinical trial evaluated the safety, efficacy, and impact on biomarkers of sequential s.c. belimumab and a single cycle of rituximab in patients with pSS. The rationale for the study was based on the complementary mechanistic effects of these 2 B cell targeting biologics and the potential for additional clinical benefits (39, 49, 50). The safety and tolerability profile of belimumab + rituximab was consistent with that of the individual monotherapies, with no new safety signals detected (21, 34, 58).

Consistent with our hypothesis, belimumab + rituximab achieved near complete depletion of CD20^+^ B cells in the MSG and a greater and more sustained depletion of peripheral CD19^+^ B cells compared with belimumab or rituximab monotherapy. Belimumab + rituximab was associated with a numerically greater improvement in some of the efficacy end points compared with placebo, including total ESSDAI score, proportion of ESSDAI responders, and stimulated salivary flow.

The study population had evidence of moderate-to-severe disease activity at baseline (ESSDAI ≥ 5), consistent with the patient selection criteria. The demographic and baseline disease characteristics were generally similar for patients in each treatment group and within expectation for patients with moderate-to-severe pSS.

The overall safety of belimumab, rituximab, and belimumab + rituximab sequential treatment in this study was consistent with the known individual safety profiles for belimumab and rituximab (21, 34, 58). Although the safety profile of belimumab + rituximab in pSS has not been explored widely, this combination has previously demonstrated acceptable safety profiles in case studies of patients with pSS (50, 57), as well as in previously published phase II studies of patients with SLE or LN, which are consistent with the results shown in this study (59–61).

Overall, there were no imbalances of clinical concern in the incidence of reported AEs or AEs of special interest (AESIs; malignant neoplasms, PASR, infections, depression, suicide/self-injury) across treatment groups. The system organ class with the highest incidence of AEs, drug-related AEs, and SAEs was infections and infestations. Given the B cell–depleting effects of both belimumab and rituximab (17, 52–54), an increase in infections might be expected with the sequential treatment relative to the monotherapies; however, this was not the case with infection and infestation AEs. Infection and infestation SAEs were reported in 4 patients in the active treatment groups compared with none in the placebo group. No serious infections of special interest were detected in any treatment group. A slightly higher incidence of depression was observed in the belimumab and belimumab + rituximab groups compared with placebo, which is consistent with the known safety profiles of belimumab and rituximab (19, 20, 25, 26). Additionally, there were 2 cases of clinical serum sickness disease in the belimumab + rituximab and rituximab groups, a rare but known side effect of rituximab (62).

Immune-complex deposition and lymphocytic infiltration, which are a result of the characteristic B cell hyperactivity observed in pSS, can result in extraglandular manifestations (3). Therefore, a change in the number of B cells can be a useful indicator of pSS pathology. The present study demonstrates a biphasic B cell response, where peripheral B cells initially rapidly increased within 1 week of belimumab administration, followed by a decrease within 8 weeks of continuous BLyS neutralization. Tabalumab, another anti-BlyS mAb treatment, demonstrated a similar biphasic response in total B cells in SLE, possibly due to disruption of B cell trafficking (63, 64). In the current study, whereas B cell subsets (such as naive B cells) decreased within 8 weeks of belimumab treatment, memory B cells appeared to be spared. Despite BLyS neutralization, memory B cells remained elevated in the circulation for a prolonged period of time, gradually decreasing to below baseline levels in patients treated with belimumab. Similar increases have been recorded in clinical trials of treatments for other autoimmune diseases; the treatments include 2 BLyS inhibitors (atacicept and blisibimod) and tabalumab (65–67). Importantly, the study presented here is the first to our knowledge to demonstrate belimumab-induced increases in peripheral memory B cells as early as week 1 after dosing. However, it remains to be demonstrated that the belimumab-induced increase in peripheral memory B cells is due to mobilization of tissue-resident B cells into the circulation and/or the prevention of circulating cells entering the tissues. Interestingly, although it was previously demonstrated that the combination of both BLyS and CXCL13 is required to attract memory B cells within the tissue, CXCL13 serum levels decreased with belimumab + rituximab treatment in this study (68). Belimumab treatment, thus, induced a pharmacodynamic window for subsequent rituximab treatment for an immediate and marked reduction in peripheral memory B cells as well as other CD20^+^ B cell subsets. Consequently, belimumab + rituximab displayed near complete depletion of total B cells, including memory B cells and other B cell subsets in the circulation, as measured by flow cytometry. The belimumab + rituximab group also showed a trend toward delayed repopulation of total B cells, with median levels of total B cells only returning to a similar level as the monotherapy groups at week 52, following belimumab discontinuation at week 24.

Analysis of serological biomarkers shows that, while serum BLyS peaked at week 12 in response to rituximab treatment, consistent with previous studies (40–42), this increase was not observed until after discontinuation of belimumab (week 24) in the belimumab + rituximab group. BLyS neutralization not only compromises B cell proliferation and survival, but it also modulates tissue-residency of B cells, as evidenced by increased levels of circulating B cells following belimumab treatment. This, in turn, may render B cells more susceptible to rituximab-mediated depletion since tissue-resident B cells are relatively more resistant to depleting effects of rituximab than those in circulation (43–45). Furthermore, it has been shown that BLyS neutralization can restore B cell susceptibility to rituximab-induced NK cell killing in allogeneic and autologous experimental systems, possibly through NK cell–derived BLyS, which enhances the metabolic activity of target cells (48). Additionally, a more sustained reduction in CXCL13 was observed in the belimumab + rituximab group. Since this chemokine facilitates the repopulation and migration of B cells into ectopic GCs, this finding is also indicative that belimumab treatment assists rituximab-induced depletion of B cells. This is further supported by the observed decrease of MSG-resident B cells at week 24. Finally, there was also a trend toward reduction in biomarkers of pSS disease activity, including IgA, IgG, IgM, RF, and serum κ and λ light chain levels.

The formation of ectopic GCs in the salivary gland is characteristic of pSS and is correlated with systemic manifestations and risk of lymphoma (3, 4). Therefore, histological assessment of salivary glands for B cells and biomarkers of B cell hyperactivity are important in monitoring pathology and predicting clinical outcome. In the present study, a near-complete depletion of MSG-resident CD20^+^ B cells was observed in the belimumab + rituximab group versus the other groups at week 24. Consistent with the peripheral memory B cell results, MSG-resident memory B cells were spared with belimumab treatment and were only slightly reduced with rituximab. However, sequential belimumab + rituximab treatment increased the depth of B cell depletion in tissue compared with belimumab, rituximab, or placebo groups. These results suggest that sequential belimumab + rituximab treatment is required to sufficiently decrease levels of tissue-resident memory B cells, which were not persistently mobilized by belimumab alone. It is important to note that, due to technical difficulties with the CD27 stain in 1 patient, only 11 patients were assessed for memory B cells in the rituximab group. This impacted the median values substantially, to give the rituximab arm the appearance of near complete depletion of memory B cells at week 24, whereas the CD20^+^ B cell counts remained relatively high at week 24.

Baseline MSG samples were less organized in the belimumab + rituximab group compared with other groups and were characterized by a lower number of mature B cells and GCs. This could suggest that the deeper depletion observed in these samples might have been favored by the lower degree of organization of baseline infiltrates, and it warrants further investigation in larger trials. Unfortunately, it is unclear from the data whether there was also an impact of treatment group on size of foci and whether this might have influenced the response. Of note, the disappearance of parotid B cell lymphoma of MALT, followed by very long–term remission, was observed previously in a patient with pSS treated with belimumab followed by rituximab (50, 51). Likewise, 3 patients with severe pSS and refractory cryoglobulinemic vasculitis, still active after anti-CD20 treatment, were successfully treated by following up the anti-CD20 treatment with belimumab (57). These data support the hypothesis that sequential belimumab + rituximab treatment depletes tissue B cells more effectively and may be more clinically efficient than belimumab or rituximab treatment alone (51, 69). Of note, despite the complete depletion of B cells in the MSG with belimumab + rituximab, the plasma cells were not affected by this combination. Since plasma cells are part of the tissular and cellular signature of the disease, the fact that plasma cells were not affected may represent a limitation of this innovative sequential treatment (3). In addition, although the lowest LFS were observed in the belimumab + rituximab and rituximab groups, the impact of sequential belimumab + rituximab treatment on LFS must be interpreted with caution, since the greatest reduction from baseline was observed in the placebo group. However, since this decrease in LFS (an indicator of the number of inflammatory foci) correlates with a decrease in the ratio of total aggregate area/total glandular area (an expression of the size of aggregates relative to overall glandular tissue), this indicates a decrease in the relative abundance of lymphoid aggregates. The decrease in these parameters is to be expected based on the decrease in B cells observed, since, where B cells are present, they are almost exclusively found in lymphoid aggregates (70).

A trend toward improvement in ESSDAI with belimumab + rituximab was observed relative to placebo and belimumab alone. The mean ESSDAI scores (total score and proportion of responders) through to week 68 consistently favored the belimumab + rituximab group versus placebo. Of note, the ESSDAI score improvement with belimumab + rituximab was sustained until week 68, despite the substantial period of time after cessation of active treatment (44 weeks), and met the 3-point threshold for a minimal clinically important improvement relative to placebo at week 68 (71). Following on from these findings, it would be interesting in future research to continue belimumab treatment in the belimumab + rituximab group until low disease activity is achieved. In addition to improvements in ESSDAI score, stimulated salivary flow at all time points to week 68 also showed a trend toward improvement in belimumab + rituximab versus either placebo, belimumab, or rituximab groups; stimulated salivary flow at baseline was higher with belimumab + rituximab compared with other groups. A previous clinical trial reporting the efficacy of ianalumab in patients with pSS also observed similar improvements in ESSDAI and stimulated salivary flow after 24 weeks of treatment (24). Interestingly, the mechanism of action of ianalumab is similar to the combination of belimumab + rituximab since it induces both B cell depletion and BLyS receptor pathway inhibition (24). In the current study, there were no notable treatment differences in oral dryness or patient-reported outcomes for any active treatment groups versus placebo.

The hypothesis that sequential belimumab + rituximab treatment may result in an improved clinical response has been supported by controlled, randomized clinical trials in patients with SLE and several small case studies (50, 55–57, 60, 72). One case study in particular reported long-term efficacy and safety of belimumab + rituximab in pSS (50, 51). In addition, combination treatment of rituximab and belimumab in the SynBioSe clinical trial led to specific reductions in anti-nuclear antibodies and neutrophil extracellular trap formation; combination treatment also achieved an acceptable safety profile, a reduction in SLE disease activity, positive renal responses, and immunosuppressive medication tapering (59). In the BEAT-LUPUS study, significant reductions in IgG anti–double-stranded DNA antibody levels and prolonged time to severe flare were also observed with combination treatment versus rituximab monotherapy in patients with SLE (60). The randomized controlled trial presented here is the first to study the sequential administration of these 2 complementary therapies in patients with pSS.

This study has several limitations to consider. This is an exploratory proof-of-mechanism study, which was not formally powered to detect differences in clinical efficacy, but a sufficient sample size was selected that would enable a reasonable evaluation of the impact of treatment on the underlying immunological mechanism and allow an exploratory assessment of efficacy. However, similarly to other recently reported trials in pSS, we observed a high level of ESSDAI placebo response (34, 73). Continued efforts are ongoing in the field to identify the most appropriate efficacy end point for trials in pSS, as there is a concern that ESSDAI does not fully capture all important elements of the burden of the disease. Work is ongoing to develop new composite end points, including the CRESS and the Sjögren’s Syndrome Tool for Assessing Response (STAR) (NECESSITY consortium) (37, 74). Such new endpoints may prove useful in future studies aimed at developing new treatments for patients with pSS. A recent post hoc analysis of the current study assessed CRESS outcomes, and treatment with belimumab + rituximab was generally associated with a numerically higher concise CRESS response rate compared with monotherapies at week 24 (52.9% belimumab + rituximab, 36.8% belimumab, 31.3% rituximab), week 52 (58.8% belimumab + rituximab, 42.1% belimumab, 25.0% rituximab), and week 68 (35.3% belimumab + rituximab, 36.8% belimumab, 18.8% rituximab) (75). However, the placebo response for CRESS was notable (week 24, 50.0%; week 52, 50.0%; week 68, 12.5%) and similar to the placebo response for ClinESSDAI. As such, the recently developed STAR could allow the placebo effect to be decreased. Finally, the imbalance in baseline median percentage of foci containing GCs across the treatment groups and the large variation in baseline MSG LFS and total B cell count in this study could impact interpretation of the results.

In conclusion, the results presented support the hypothesis that anti-BLyS and anti-CD20 therapies act in a mechanistically complementary manner in pSS and may represent a novel treatment approach, if validated by larger studies powered to demonstrate improved clinical efficacy relative to the individual monotherapies. In particular, rituximab decreases peripheral memory B cells, which are usually spared by belimumab monotherapy in pSS. In a similar way, the limited efficacy of rituximab monotherapy in pSS could be overcome by belimumab-induced depletion of tissue-resident B cells and the inhibition of postrituximab serum BLyS increase, which usually favors the reemergence of autoreactive B cells. This sequential treatment approach may also be relevant in other autoimmune diseases where rituximab alone has transient or limited efficacy.

## Methods

Supplemental Methods are available online with this article.

### Study design.

This phase II study (GSK study 201842, NCT02631538) comprised a randomized, double-blind, placebo-controlled 52-week treatment period and a 16-week follow-up period. Patients who continued to have B cell levels below the lower limit of normal after completion of the 16-week follow-up period had the option to enter an additional 36-week individualized follow-up period (Figure 1).

### Patients.

Eligible patients were ≥ 18 years of age with a documented diagnosis of pSS (according to American-European Consensus Group criteria) (76), active systemic disease (ESSDAI score ≥ 5 at screening) (71), symptomatic oral dryness (patient-completed Numeric Response/Rating Scale [NRS] ≥ 5/10) and unstimulated salivary flow > 0.0 mL/min or evidence of glandular reserve function at baseline (stimulated salivary flow > 0.05 mL/min) at baseline. Full eligibility criteria, including exclusion criteria and medication and laboratory parameter restrictions, are in the Supplementary Methods.

The safety population included all patients who received ≥ 1 dose of study treatment. The completer population included patients who completed the 52-week treatment and 16-week follow-up periods (including the study visit at week 68) and excluded patients who prematurely discontinued study treatment.

### Interventions.

Patients were randomized (1:2:2:2) to 1 of 4 treatment arms; placebo (belimumab placebo s.c. weekly to week 51, with rituximab placebo i.v. infusions at weeks 8 and 10), sequential belimumab + rituximab (belimumab 200 mg s.c. weekly to week 24 followed by belimumab placebo s.c. weekly to week 51, with rituximab 1,000 mg i.v. at weeks 8 and 10), belimumab monotherapy (belimumab 200 mg s.c. weekly to week 51, with rituximab placebo i.v. at weeks 8 and 10), or rituximab monotherapy (belimumab placebo s.c. weekly to week 51, with rituximab 1,000 mg i.v. at weeks 8 and 10) (Figure 1 and Supplementary materials). For patients in the sequential belimumab + rituximab treatment arm, belimumab therapy was discontinued at week 24 to determine if clinical, functional, and mechanistic treatment effects may be sustained after discontinuation of therapy until week 52. In particular, it was of interest to determine whether B cell repopulation would occur and at what time point following discontinuation. Except for a pharmacist who prepared the i.v. rituximab infusions, all study site personnel, patients, and the sponsor’s study team remained blinded to the study agent received (Supplementary Methods). Patients were stratified by screening ESSDAI scores (5–12 versus >12). Use of concomitant biologic treatments, conventional systemic immunosuppressive treatments and disease-modifying antirheumatic drugs (such as methotrexate and azathioprine), pharmacological topical ophthalmic agents (such as nonsteroidal antiinflammatory drugs, corticosteroids, cyclosporine, and diquafosol), and nonmuscarinic secretagogues (such as anetholtrithione, bromhexine, and N-acetylcysteine) was prohibited during the study.

### Outcomes.

Endpoints are presented as safety outcomes, immunological outcomes, and clinical outcomes; however, the end point hierarchy is presented in Supplemental Table 5. For all end points, baseline was defined as day 0. If a patient’s day 0 value was missing, the screening value was used as baseline.

The primary end point was safety to week 68, assessed in the safety population, including incidence of AEs and AESIs. AESIs included malignant neoplasms, PASR, all infections of special interest (opportunistic infection, herpes zoster, tuberculosis, sepsis), depression/suicide/self-injury, and deaths, as well as study-specific AESIs of severe skin reactions, cardiac disorders, posterior reversible encephalopathy syndrome, progressive multifocal leukocephalopathy, and biopsy-related AEs.

Immunological end points were assessed in both the safety and completer populations and are presented here for the completer population. They include number of B cells (total [CD19^+^], memory [CD20^+^CD27^+^], naive [CD20^+^CD27^–^], and plasmablast [CD27br^+^CD38br^+^CD19^+^]) measured by flow cytometry to week 68, change in serological biomarkers (IgG, RF, IgA, IgM, free BLyS, total BLyS, C3, C4, CH50, κ and λ light chain, κ/λ ratio, β2 microglobulin, CXCL13, SS-A, SS-B) over time, MSG CD20^+^ B cells at baseline and week 24, change in histological assessments of salivary gland biopsy samples at baseline versus week 24, and change in MSG biomarkers (LFS, B cells, B cell/T cell ratio, plasma cells, total aggregate area/total glandular area ratio, average focus size, foci displaying GCs, foci displaying follicular DCs, foci displaying CD3/CD20 segregation, plasma cell/B cell ratio, memory B cells [switched and nonswitched], follicular B cells) over time.

Clinical end points were assessed in both the safety and completer populations and are presented here for the completer population. They include mean ESSDAI total score over time to week 68, the proportion of ESSDAI responders to week 68 (category 1, ≥ 3-point improvement in total ESSDAI versus baseline; category 2, ≥ 5-point improvement in total ESSDAI versus baseline; category 3, ESSDAI total score <5 ), the proportion of ClinESSDAI responders to week 68 (ClinESSDAI total score < 5), mean stimulated salivary flow over time to week 68, and oral dryness NRS to week 68, mean ESSPRI over time to week 68 by total score and domain (dryness, fatigue, and pain), and changes from baseline in lacrimal gland function (Schirmer’s test) and unstimulated salivary flow. The ESSDAI is a systemic disease activity index designed to measure systemic disease activity in pSS, and the ESSPRI is a disease-specific patient-reported index designed to assess the severity of patients’ symptoms in pSS (77, 78).

### Flow cytometry.

B cell flow cytometry panels were used to measure changes in the total B cell, naive, memory, and plasma B cell compartments over the course of treatment. The gating strategy for the flow cytometry analysis can be found in the flow cytometry gating strategy section of the Supplementary Methods.

Following completion of the study and database freeze, a small number of errors was identified in the B cell flow cytometry data, caused by manual data entry. Specifically, of the approximately 2,600 patient-level values that contribute to the displays in Figures 1 and 6 (0.2%), incorrect values were included in the median and interquartile range calculations. The sponsor’s assessment is that the 6 incorrect values had no effect on the overall interpretation or inferences drawn from the flow cytometry data reported in this manuscript.

### MSG histology.

Histological analysis of salivary gland biopsy samples (lymphocyte infiltrate, B cell, and T cell subsets) was performed through analysis of foci scoring and IHC. IHC assessments included B cell and T cell markers. MSG biopsies from screening and week 24 after treatment were formalin fixed and paraffin embedded according to routine laboratory procedures. Histological analysis of H&E staining was performed on 3 μM sections taken from 2 separate cutting levels that were 100 μm apart. Stained sections were digitally imaged using a Leica Aperio AT2 digital slide scanner (Leica Biosystems) and analyzed by trained analysts using Leica Slidepath software (v4.0.7). Routine analysis included the calculation of the LFS (the number of lymphocytic aggregates per 4 mm^2^ glandular tissue), the average focus size (μm^2^), and the area fraction (total lymphocytic area/total glandular tissue area).

Manual immunofluorescence staining for B cells and plasma cells were performed with primary antibodies against CD20 (Dako, L26, M0755; a clone demonstrated not to be blocked by rituximab binding; ref. 79) and CD138 (Bio-Rad, B-A38, MCA2459GA). CD20^+^ and CD138^+^ cells were quantified using Definiens Tissue Studio (Definiens AG) with which a machine learning pattern recognition-based approach is used to train the software to identify the tissue section within a digital image and segment this into distinct anatomical and cellular regions. These cellular segments are then characterized and quantified based on their relative expression profiles.

Any changes in lymphocyte populations in glandular tissue were further evaluated with epigenetic quantification or other equivalent technology, and/or additional IHC markers of leukocyte infiltration and activation and/or glandular biology. Screening salivary gland biopsies were assessed for lymphoma risk by a pathologist. All other samples were scored by trained lab staff under the supervision of, and subject to review by, a consultant rheumatologist with expertise in salivary gland/pSS histopathology.

### Serology.

Quantification of serum analytes, autoantibodies, markers of B cell activation, cytokines, chemokines, and other analytes associated with immune activation was performed using Luminex (Luminex Corporation), ELISA, or other appropriate technologies on serum.

### Data sharing.

Anonymized individual participant data and study documents can be requested for further research at http://www.clinicalstudydatarequest.com

### Prior presentation.

A portion of the data presented in this manuscript was presented as an oral presentation at the EULAR 2021 virtual congress on June 2–5, 2021 (80), and as a poster presentation at the American College of Rheumatology (ACR) convergence 2021 virtual congress on 1–10 November 2021 (81).

### Statistics.

Approximately 70 patients were planned for inclusion (see sample size and statistical methods section of Supplemental Methods). Therefore, 86 patients were enrolled to account for the potential withdrawal of several patients throughout the study. No formal statistical comparisons were made on efficacy and other end points (Supplementary methods).

### Study approval.

Written informed consent was obtained from each patient. The study protocol (82), amendments, and informed consent form were reviewed and approved by a national, regional, or investigational center ethics committee or IRB, in accordance with the International Conference on Harmonisation of Technical Requirements for Registration of Pharmaceuticals for Human Use (ICH) Good Clinical Practice (GCP) and applicable country-specific requirements. The IRBs included: Comite de Etica en Investigacion Clinica — CEIC, Buenos Aires, Argentina; Comité de Ética Instituto Reumatologico Strusberg, Córdova, Argentina; University Health Network, Research Ethics Board, Ontario, Canada; Advarra, Ontario, Canada; CPP Ile-de-France XI, Saint-Germain-en-Laye Cedex, France; Ethikkommission der Universität zu Lübeck, Lübeck, Germany; Comitato Etico per la Sperimentazione dell’Azienda Ospedaliera di Padova, Padova, Italy; St. Antonius Ziekenhuis, Nieuwegein, Netherlands; REK, Regionale komiteer for medisinsk og helsefaglig forskningsetikk, Oslo, Norway; Hospital la Paz, Madrid, Spain; Regionala etikprövningsnämnden I Lund, Lund, Sweden; and North East Newcastle and North Tyneside 2 Research Ethics Committee, Newcastle upon Tyne, United Kingdom. The study was conducted in accordance with GCP and the Declaration of Helsinki.

## Author contributions

XM, FB, HB, KLC, SDV, RBH, MH, RP, RS, JS, AVM, NW, DAR, and PPT contributed to the conception or design of the study. XM, CB, SDV, KL, RS, and PLAVD contributed to the acquisition of the data. XM, FB, CB, HB, KLC, SDV, DHG, RBH, KL, PM, RP, RS, PLAVD, AVM, NW, DAR, and PPT contributed to the analysis or interpretation of the data.

## Supplementary Material

Supplemental data

ICMJE disclosure forms

## Figures and Tables

**Table 1:**
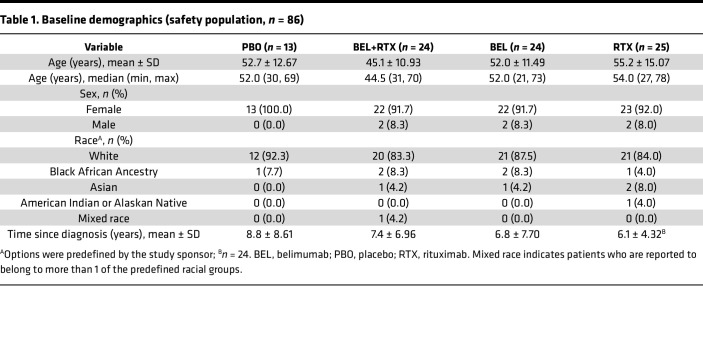
Baseline demographics (safety population, *n =* 86)

**Table 2:**
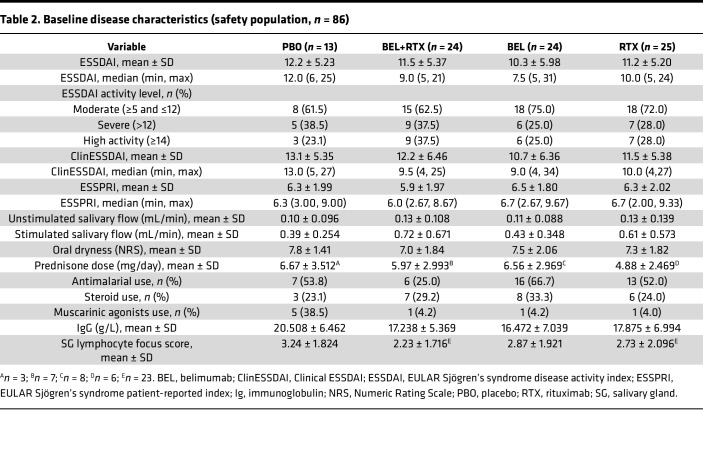
Baseline disease characteristics (safety population, *n =* 86)

**Table 3:**
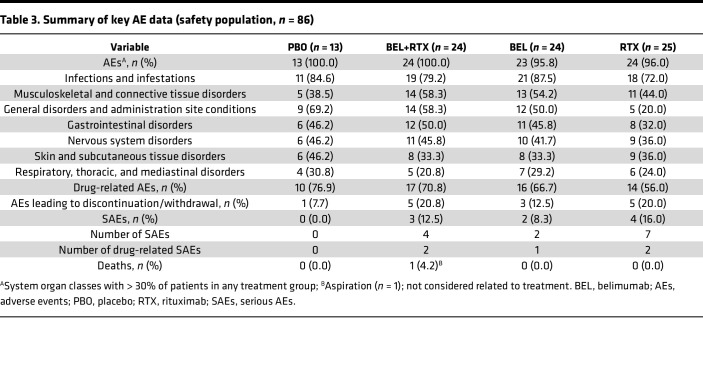
Summary of key AE data (safety population, *n =* 86)

**Table 4:**
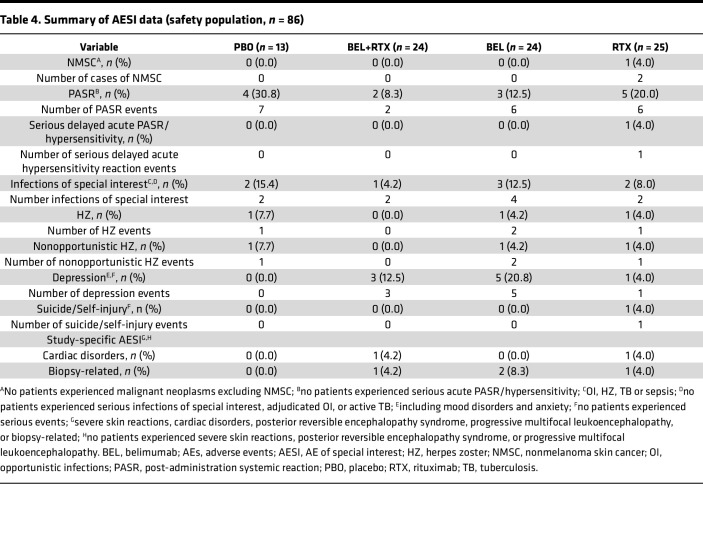
Summary of AESI data (safety population, *n =* 86)

## Version 1 12/08/2022

Electronic publication
