# Supplementary material for: A randomized, phase II study of sequential belimumab and rituximab in primary Sjögren’s syndrome
Source: JCI Insight. 2022 Dec 8;7(23):e163030. doi: 10.1172/jci.insight.163030 (PMC9746921; doi:10.1172/jci.insight.163030)
Supplement: Supplemental data [file jciinsight-7-163030-s237.pdf]

SUPPLEMENTARY MATERIALS

Supplementary methods

Study design

Study was conducted at 31 sites in 10 counties (Argentina, Canada, France, Germany, Italy, Netherlands, Norway, Spain, Sweden, United Kingdom).

Exclusion criteria

- Diagnosis of secondary Sjögren's syndrome
- Active life-threatening or organ-threatening complications of Sjögren's syndrome disease (based on treating physician evaluation)
- Severely immunocompromised state
- Severe cardiac disease
- Liver disease (with the exception of Gilbert's syndrome or asymptomatic gallstones)
- History of major organ transplant (including hematopoietic stem cell transplant), malignancy within the past 5 years, acute or chronic infections requiring management, or hypersensitivity to belimumab and/or rituximab or to other mAbs
- Progressive multifocal leukoencephalopathy (PML)
- Risk of suicide (based on investigator's judgement or history of attempted suicide/suicidal ideation over the 6 months prior to screening)
- Patients were also excluded if they had used systemic immunosuppressive or immunomodulatory agents within 60 days prior to the study, cyclophosphamide within 180 days prior to the study, anti-BLyS, anti-CD20, anti-CD22 or anti-CD52 or any other B-cell depleting agent within 364 days prior to the study, abatacept or any biologic agent within 180 days prior to the study, IVIG or plasmapheresis within 90 days prior to the study, oral steroid >10 mg prednisone equivalent/day within 30 days prior to the study or oral steroid >20 mg prednisone equivalent/day for a minimum

of 2 consecutive weeks within 60 days prior to the study, parenteral steroid within 60 days prior to the study or a live vaccine within 30 days prior to the study.

- IgA deficiency (IgA level <10 mg/dL)
- Any of the following screening laboratory values:
    - White blood cells <1 x 10^9/L
    - Neutrophils <1.5 x 10^9/L
    - Circulating IgG <550 mg / dL
    - Aspartate aminotransferase (AST) >2.0 times the upper limit of normal (ULN)
    - Alkaline phosphatase (ALP) >1.5 times the ULN
    - Bilirubin >1.5 times the ULN (unless direct bilirubin fraction is <35%)
    - CD 19+ B-lymphocyte counts <0.1 x 10^9/L (<100 per CMM) (applies only to patients previously exposed to B-cell depleting therapies)
- Patients were withdrawn from study treatments if any of the following criteria were met:
    - IgG <400 mg/dL or neutrophil count <1x10^9/L, confirmed by repeat test 1 week (>2 days) after the initial result
    - Decrease in IgG to <550 mg/dL associated with a serious infection
    - Life-threatening infection (regardless of IgG status)
    - Confirmed PML
    - Severe skin reactions (believed to be treatment-related)
    - Liver stopping criteria
    - Suicide risk
    - Positive pregnancy test

Treatment randomization and blinding

An unblinded member of staff at each study site was assigned to use the randomization software (RAMOS NG), and receive drug shipments and notifications. An unblinded pharmacist at each study site prepared rituximab and its placebo for IV administration. Unblinded monitors were assigned to review all pharmacy records, storage and procedures. The Internal Safety Review Committee (who

unblinded safety and efficacy data during the study, including an interim analysis, to ensure patient safety and allow for sample size adjustment) and therapeutic area data assessment committee were unblinded to safety and efficacy data but contact between Internal Safety Review Committee members and investigators was prohibited. During treatment and follow-up, all other study staff remained blinded to treatment allocation and central laboratory data that had the potential to unblind treatment assignment. Treatment codes could be unblinded by the investigator or treating physician only in the case of a medical emergency or in the event of a serious medical condition. GSK Global Clinical Safety and Pharmacovigilance staff could unblind treatment codes in the event of a SAE.

Sample size and statistical methods

This study was not powered to detect pre-defined treatment differences. Approximately 70 patients were planned for inclusion, with 20 randomized to each treatment group and 10 randomized to placebo. Since the primary objective of the study was to investigate safety and tolerability, no formal statistical hypothesis testing was planned. Additionally, no formal statistical comparisons were made on secondary efficacy and other mechanistic endpoints as the study was not sized based on statistical power considerations. All data summaries and analyses were performed using the latest available version of Statistical Analysis System (SAS Institute Inc., Cary, NC, USA) software (Version 9.4).

For key exploratory and health outcome endpoints, descriptive statistics of the absolute values and change from baseline were used to summarize the endpoints by treatment group and visit. Change in flow cytometry and salivary gland parameters were analyzed using the Hodges-Lehmann method to provide a non-parametric 95% confidence interval (CI) for the treatment comparisons of interest. For the ESSDAI responder analyses utilized a generalized estimating equation (GEE) model.

The secondary endpoint of minor salivary gland CD20+ B cell absolute count/mm^2 at baseline versus Week 24 was analyzed using the Hodges-Lehmann method to provide a 95% CI for treatment comparisons of interest. Other secondary endpoints were analyzed using mixed model repeated measures analysis (ESSDAI score, stimulated salivary flow, oral dryness NRS, salivary gland B-cell quantification) or GEE model (ESSDAI responder analysis).

Flow cytometry gating strategy

Immunophenotyping was performed by flow cytometry with absolute cell numbers calculated using TruCOUNT beads. Representative dot plots showing gating strategy for flow cytometry analysis of whole blood cells are shown below, labeled with antibodies to CD19, CD20, CD27, CD38, CD45 and IgD.

Three different assays were utilized to identify and enumerate the total CD19+ B cells and the subsequent B-cell subpopulations reported in this study. Data for all the assays were acquired on a BD FACSCanto instrument.

Plots 1–5: The total CD19+ B cells are derived from a standard lymphocyte subset panel which utilized BD Clinical Canto software for enumeration of T cells, B cells and natural killer (NK) cells.

Plot 1 Plot 2 Plot 3 Plot 4 Plot 5

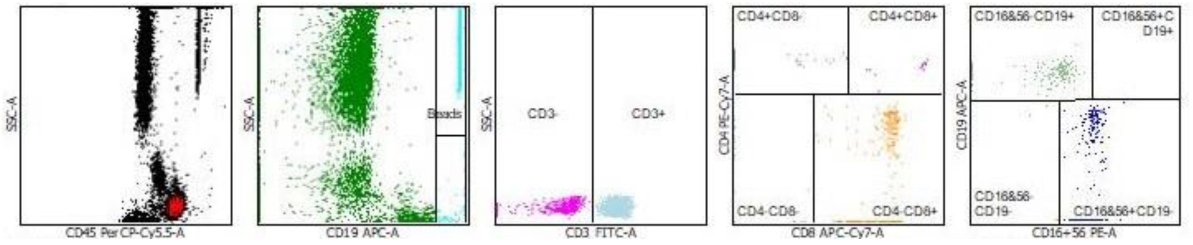

Plot 1: Lymphocyte cells identified as CD45 positive side scatter area (SSC-A)^{low} events. Plot 2: TruCOUNT bead events are captured on the SSC-A versus CD19 plot. Bead counts are used in calculation of absolute counts of each lymphocyte subset. Plot 3: Total lymphs are displayed, and T cells are gated as CD3+ events. Plot 4: From the CD3+ events, the T helper cells (CD4+CD8-) and T cytotoxic cells (CD4-CD8+) are gated with the help of quadrant gate. Plot 5: From the CD3- events quadrant gate is used to set the gates for NK cells (CD16+56+CD19-) and B cells (CD56-CD16-CD19+).

Plots 6–10: Two-tube B-cell panel consisting of CD19, CD20, CD38 and CD27 antibodies is utilized for identification of memory B cells and plasmablast cells.

Plot 6

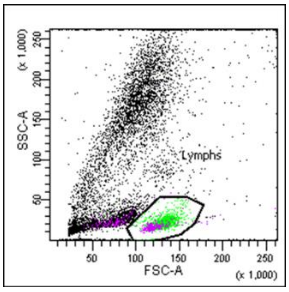

Plot 7

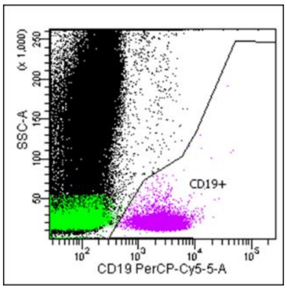

Plot 8

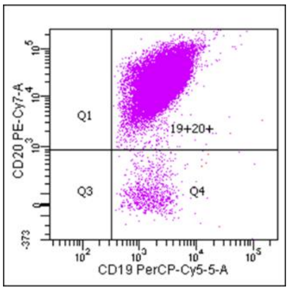

Plot 9a

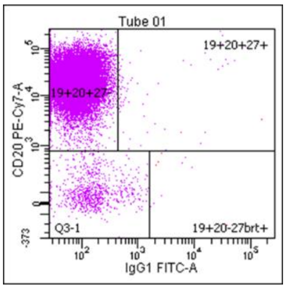

Plot 9b

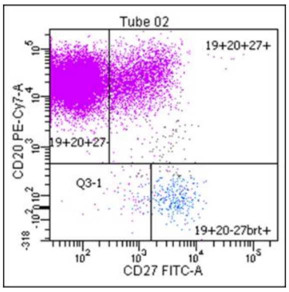

Plot 10a

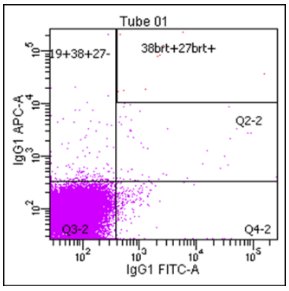

Plot 10b

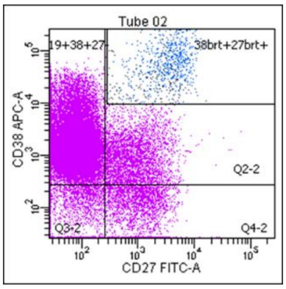

Plot 6: Gating of lymphocyte events on forward scatter area (FSC-A) versus SSC-A plot. Plot 7: Total CD19+ cells are identified as SSC-A^{low+} and CD19+ events. Plot 8: B cells are identified as CD19+CD20+ events. Plot 9: Total CD19 events are displayed on CD27 versus CD20 plot to identify the Memory B cells – gated as CD19+CD20+CD27+ events. The panel utilizes an isotype tube that is used to set the negative cut-off for CD27 gate (Plot 9a), the gate remains unchanged in the test tube (Plot 9b). Plot 10: From the total CD19 cells, plot displaying CD27 versus CD38, isotype tube is used to set the cut-off for CD27 as well as CD38 (Plot 5a). In test tube (Plot 5b), the plasmablast cells are identified as CD19+ CD38^{BRIGHT} CD27^{BRIGHT} events.

Plots 11–13: One-tube B panel consisting of CD19, CD20, CD27 and IgD antibodies is used to identify the naïve B cells.

Plot 11

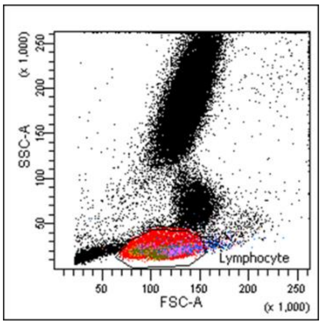

Plot 12

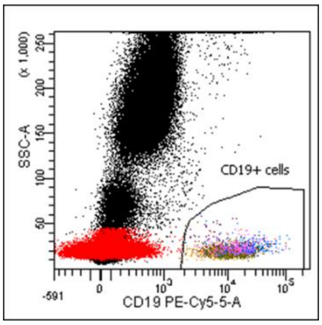

Plot 13

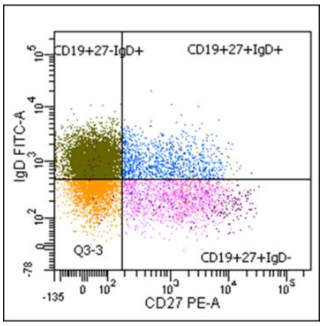

Plot 12: Gating of lymphocyte events on FSC-A versus SSC-A plot. Plot 13: Total CD19+ events identified as SSC-A^{low+} and $CD19^{High+}$. Plot 14: The naïve B cells are identified as CD19+CD27-IgD+ events using quadrant gate on CD27 versus IgD plot.

Supplementary figures and tables

Supplementary Figure 1. Median (IQR): A) switched memory B cells (CD19+ CD27+ IgD-), B) unswitched memory B cells (CD19+ CD27+ IgD+) over time by flow cytometry (completer population, N=60)

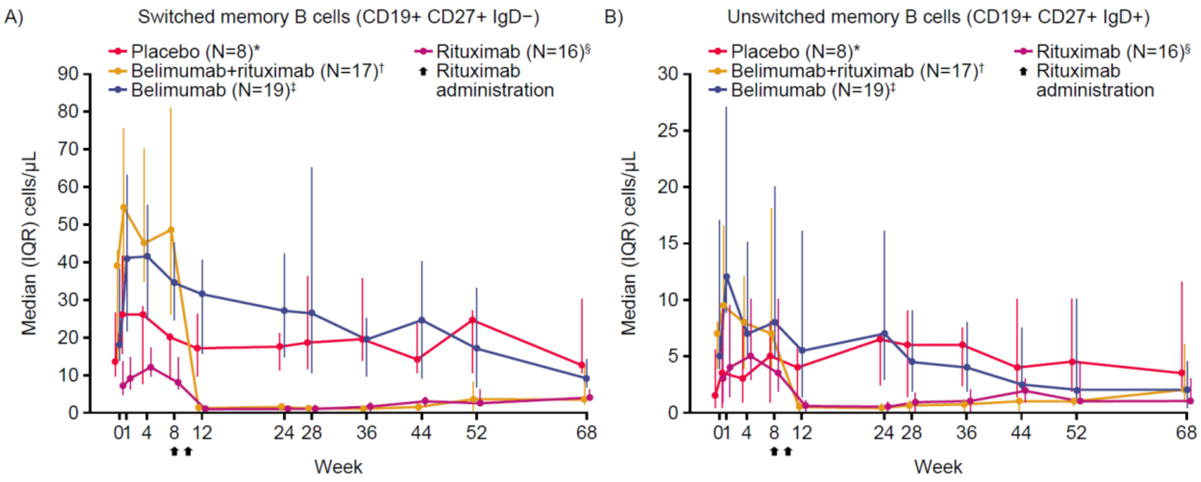

Flow cytometry data were analyzed using the Hodges-Lehmann method to provide a non-parametric 95% confidence interval for the treatment comparisons of interest.

*N=7 at weeks 4, 8, and 12. N=6 at week 52. †N=16 at weeks 1, 8, 36, 44, 52, and 68. ‡N=17 at week 1. N=18 at weeks 4, 8, 28, and 36. N=16 at weeks 12, 44, and 68. N=15 at week 52. §N=13 at week 24. N=14 at week 36. N=15 at weeks 44 and 68.

IgD, immunoglobulin D; IQR, interquartile range

Supplementary Figure 2. Median (IQR) absolute values of A) CD3+ CD4+ and B) CD3+ CD8+ T cells over time (completer population, N=60)

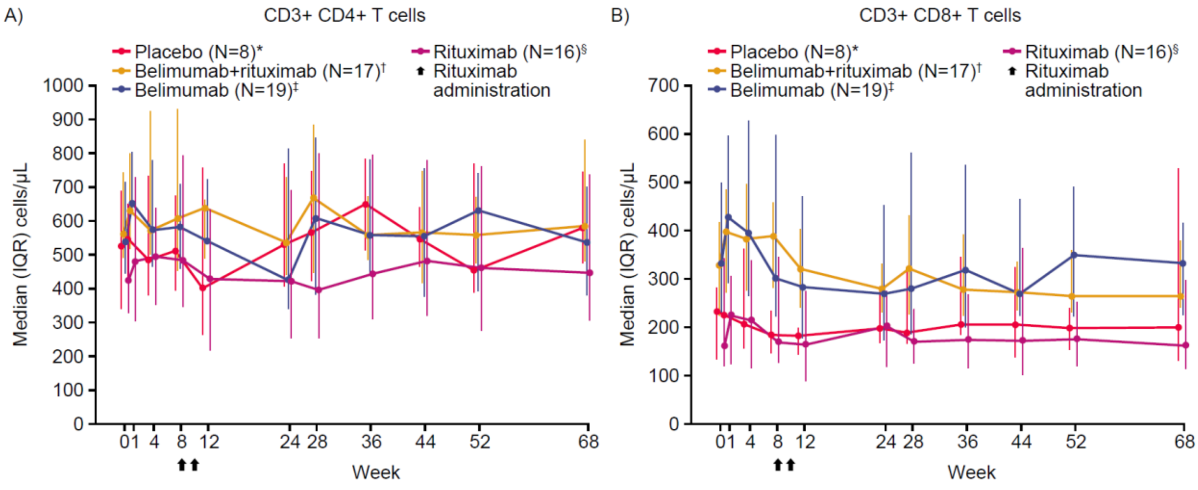

Flow cytometry data were analyzed using the Hodges-Lehmann method to provide a non-parametric 95% confidence interval for the treatment comparisons of interest.

*N=7 at weeks 4, 12, and 52. †N=16 at weeks 1, 36, 44, and 68. ‡N=17 at weeks 1, 44, and 68. N=18 at weeks 4, 12, 28, and 36. N=16 at week 52. §N=13 at week 24.

IQR, interquartile range

Supplementary Figure 3. Immunofluorescent (Hoechst CD20) and hematoxylin and eosin histological images

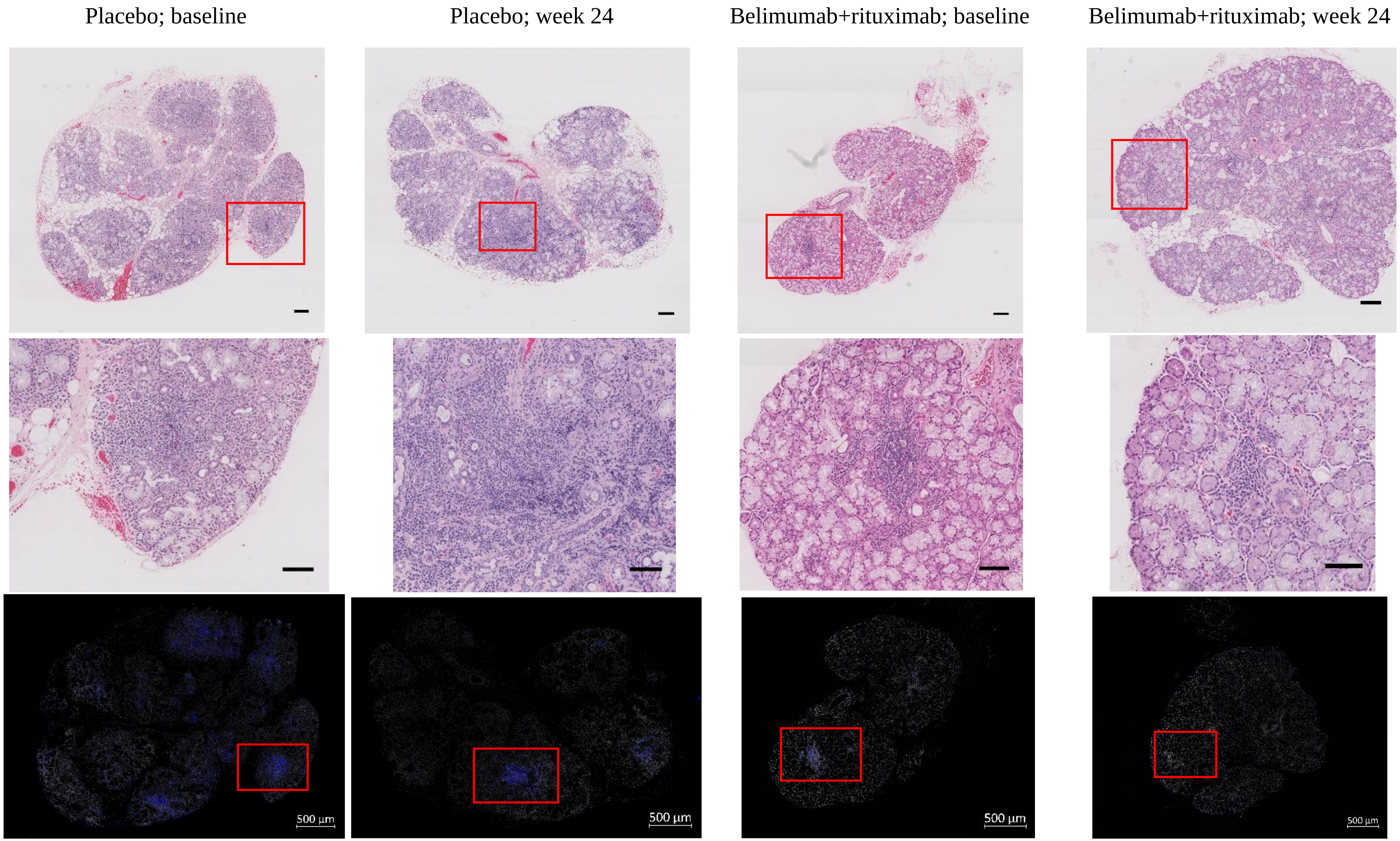

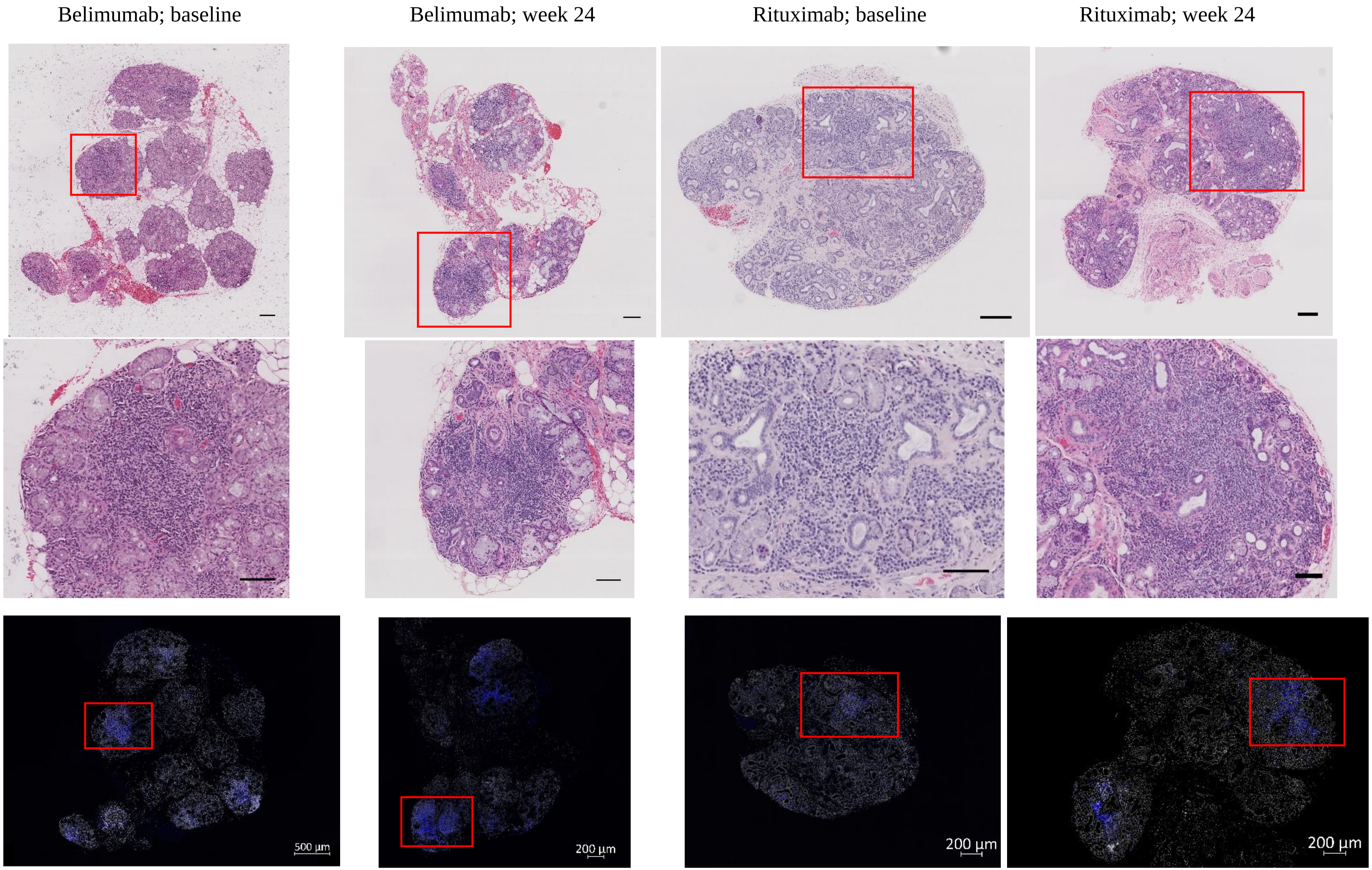
Belimumab; baseline
Belimumab; week 24
Rituximab; baseline
Rituximab; week 24
500 µm
200 µm
200 µm
200 µm

Original slides (row 1) were imaged at 20x using a Zeiss Axio Scan Z1 slide scanner. In the overall sample images (rows 1 and 3) the scale bar represents 200 µm. For the region of interest images (row 2), the scale bar is 100 µm.

Supplementary Figure 4. Clinical efficacy over time as measured by mean (SE) oral dryness (completer population, N=60)

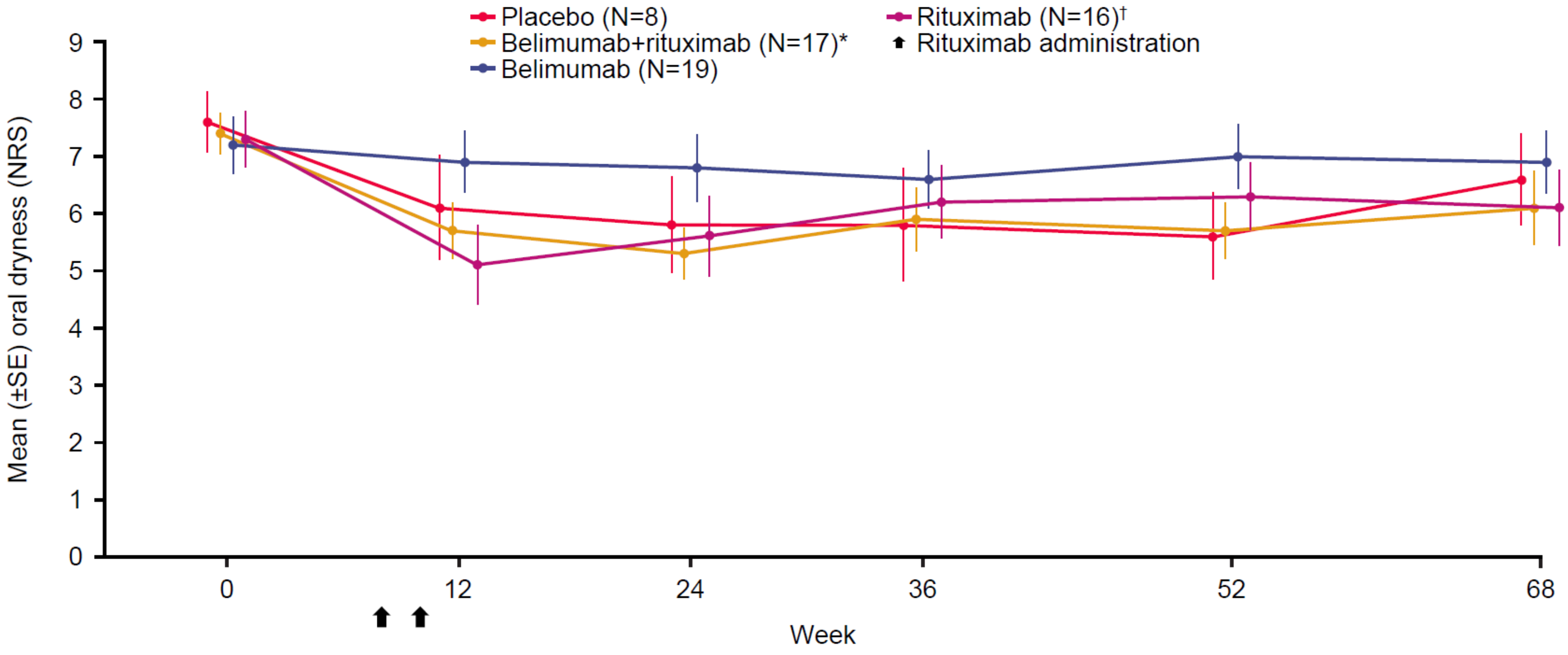

*N=16 at week 52. †N=15 at week 24.

NRS, Numeric Rating Scale; SE, standard error.

Supplementary Figure 5. Components of ESSPRI: A) dryness, B) fatigue, and C) pain domain (completer population, N=60)

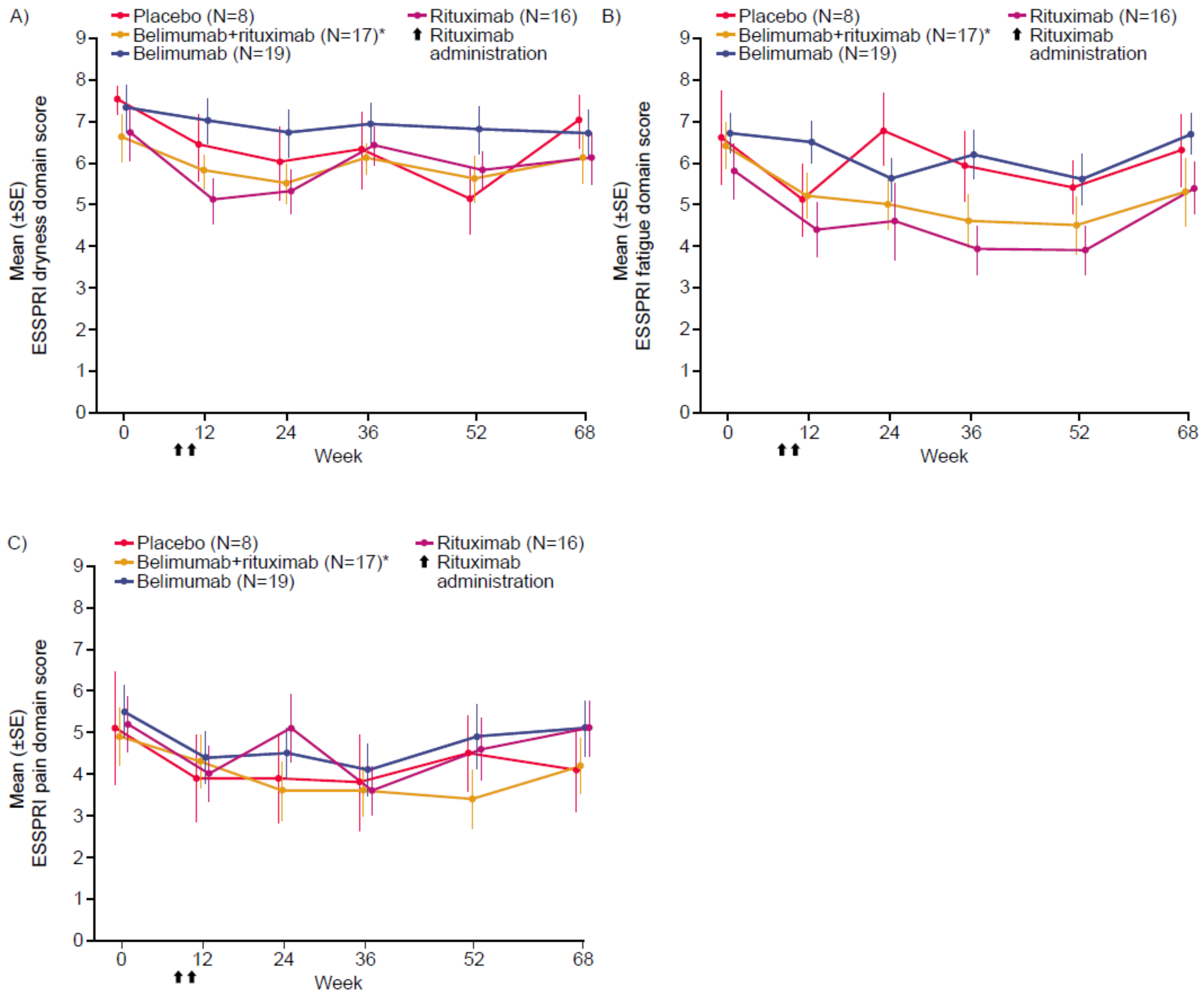

*N=16 at week 52.

ESSPRI, EULAR Sjögren's Syndrome Patient Reported Index; SD, standard deviation.

Supplementary Table 1. Infection and infestation SAEs by preferred term (safety population, N=86)

**Infection and infestation SAE by preferred term, n (%)**	**PBO (N=13)**	**BEL+RTX (N=24)**	**BEL (N=24)**	**RTX (N=25)**
Any event	0 (0.0)	2 (8.3)	1 (4.2)	1 (4.0)
Bronchitis	0 (0.0)	0 (0.0)	0 (0.0)	1 (4.0)
Enterocolitis infectious	0 (0.0)	1 (4.2)	0 (0.0)	0 (0.0)
Ophthalmic HZ	0 (0.0)	0 (0.0)	0 (0.0)	1 (4.0)
Pneumonia	0 (0.0)	0 (0.0)	1 (4.2)	0 (0.0)
Pyelonephritis	0 (0.0)	1 (4.2)	0 (0.0)	0 (0.0)

BEL, belimumab; HZ, herpes zoster; PBO, placebo; RTX, rituximab; SAE, serious adverse event.

Supplementary Table 2. Change of serum biomarkers following treatment (completer population, N=60)

**Serum biomarker**	**PBO (N=8)**	**BEL+RTX (N=17)**	**BEL (N=19)**	**RTX (N=16)**
**IgG, median (min, max) g/L**				
Baseline	18.4 (12.9, 31.3)	15.1 (10.1, 26.2)	16.2 (5.7, 32.6)	15.1 (8.5, 34.7)
Week 12	16.3 (10.6, 26.6)	13.6 (8.8, 19.9)	15.4 (5.7, 21.8)	14.6 (9.0, 31.6)
Week 24	17.4 (12.0, 26.0)	13.0 (7.9, 18.5)	14.8 (6.1, 22.4)	13.9 (8.8, 32.1)
Week 36	18.0 (11.9, 29.8)	12.4 (8.1, 21.3)	15.5 (6.1, 22.9)	14.2 (8.5, 24.4)
Week 52	17.8 (11.0, 27.4)	13.2 (8.5, 22.5)	14.5 (6.8, 20.1)	14.3 (9.5, 28.4)
Week 68	22.4 (10.4, 28.6)	13.8 (8.8, 23.0)	15.6 (6.8, 22.1)	14.1 (7.6, 37.4)
**RF, mean ± SD KU/L***				
Baseline	79.3 ± 29.0[†]	66.9 ± 36.3[‡]	48.4 ± 34.1[§]	150.5 ± 230.9[¶]
Week 12	58.9 ± 33.2	33.3 ± 17.8[†]	34.8 ± 26.8**	152.1 ± 219.6[††]
Week 24	74.4 ± 23.5[‡‡]	25.4 ± 10.2[‡‡]	39.5 ± 31.8[§§]	103.8 ± 131.4[††]
Week 36	65.8 ± 32.4[†]	31.4 ± 19.4[‡‡]	37.5 ± 24.0[§§]	104.8 ± 100.7[††]
Week 52	49.7 ± 28.4[†]	48.0 ± 35.7[‡‡]	31.0 ± 15.2[††]	113.4 ± 117.8[§§]
**IgA, median (min, max) g/L**				

Baseline	2.9 (2.5, 5.0)	3.0 (1.9, 6.7)	2.5 (0.8, 5.6)	2.6 (0.4, 4.7)
Week 12	2.9 (2.2, 5.1)	2.9 (1.7, 4.7)	2.3 (0.6, 4.3)	2.7 (0.5, 5.1)
Week 24	2.7 (2.2, 4.7)	2.6 (1.5, 4.7)	2.4 (0.6, 4.6)	2.7 (0.4, 4.8)[¶¶]
Week 36	2.9 (2.2, 4.9)	2.6 (1.7, 4.8)	2.3 (0.6, 4.3)***	2.6 (0.4, 4.3)
Week 52	2.8 (2.1, 4.5)	2.6 (1.5, 5.4)	2.2 (0.6, 4.2)	2.6 (0.4, 5.1)
Week 68	2.8 (2.3, 4.5)	2.7 (1.5, 6.5)	2.1 (0.5, 4.8)	2.6 (0.3, 4.2)
**IgM, median (min, max) g/L**				
Baseline	1.4 (0.8, 4.1)	0.9 (0.4, 2.0)	1.3 (0.3, 2.6)	1.1 (0.3, 2.1)
Week 12	1.4 (0.8, 4.3)	0.7 (0.3, 1.9)	1.1 (0.2, 2.1)	1.1 (0.3, 1.9)
Week 24	1.3 (0.6, 4.5)	0.5 (0.3, 1.3)	1.1 (0.2, 2.3)	1.0 (0.3, 1.7)[¶¶]
Week 36	1.3 (0.6, 4.4)	0.5 (0.2, 1.4)	1.1 (0.2, 2.2)***	0.8 (0.3, 1.5)
Week 52	1.4 (0.6, 4.2)	0.5 (0.3, 2.0)	1.0 (0.2, 1.9)	1.0 (0.3, 1.6)
Week 68	1.2 (0.6, 3.4)	0.5 (0.3, 2.3)	0.9 (0.2, 1.9)	0.8 (0.3, 2.0)
**Free BLyS, median (IQR) ng/mL**				
Baseline	1.1 (0.8–1.4)	0.8 (0.6–1.2)	0.6 (0.6–0.9)	1.1 (1.0–1.4)
Week 12	0.9 (0.8–1.4)	0.1 (0.0–0.2)	0.1 (0.0–0.4)	3.9 (2.3–5.4)
Week 24	1.1 (0.6–1.9)	0.1 (0.1–0.2)	0.1 (0.0–0.1)	3.4 (1.9–6.1)

Week 36	1.1 (0.7–1.4)	1.1 (0.4–2.7)†††	0.1 (0.0–0.1)	2.1 (1.6–3.7)
Week 52	1.0 (0.8–1.8)	2.7 (1.1–3.6)	0.1 (0.0–0.1)	1.6 (1.0–2.4)
Week 68	0.9 (0.7–1.6)	1.7 (1.3–3.1)	1.4 (0.9–1.7)	1.4 (0.9–3.1)
**Total BLyS, median (IQR) pg/mL**				
Baseline	840.7 (607.0–1653.5)	675.3 (579.3–842.4)	560.9 (444.5–801.8)***	736.1 (603.3–1140.5)¶¶
Week 12	756.7 (584.9–938.1)	20189.0 (17592.3–24743.0)†††	21470.0 (17877.8–27301.3)	2410.1 (1413.8–3836.9
Week 24	880.1 (596.8–1238.6)	21095.2 (17799.1–26730.8)	20485.5 (15636.1–29557.1)	2490.3 (1630.2–3948.6)
Week 36	671.8 (535.4–882.2)	33362.6 (22076.6–57574.9)†††	23220.4 (18719.4–28900.8)	1696.6 (1403.7–3673.5)
Week 52	720.9 (670.7–1198.8)	6052.1 (2511.9–13475.7)	23197.6 (15141.6–29686.4)	1074.9 (786.2–2663.0)
Week 68	1062.6 (731.4–1587.7)	1670.5 (1254.9–2014.0)	18404.9 (6526.3–48745.2)	1325.8 (935.0–1999.4)
**Complement component 3, median (IQR) g/L**				

Baseline	1.3 (1.1–1.4)	1.2 (1.1–1.3)	1.0 (1.0–1.4)	1.0 (1.0–1.2)
Week 12	1.2 (1.1–1.5)	1.2 (1.1–1.3)†††	1.1 (0.9–1.3)	1.1 (1.0–1.2)
Week 24	1.2 (1.1–1.3)	1.3 (1.1–1.3)	1.1 (1.0–1.3)	1.1 (1.0–1.2)¶¶
Week 36	1.2 (1.1–1.4)	1.2 (1.1–1.3)†††	1.1 (0.9–1.3)	1.2 (1.0–1.4)
Week 52	1.3 (1.0–1.4)	1.3 (1.0–1.4)	1.1 (0.9–1.3)	1.1 (1.0–1.2)
Week 68	1.2 (1.0–1.5)	1.2 (1.1–1.3)	1.1 (0.8–1.4)	1.1 (1.0–1.3)
**Complement component 4, median (IQR) g/L**				
Baseline	0.2 (0.1–0.2)	0.2 (0.2–0.2)	0.2 (0.2–0.2)	0.2 (0.1–0.2)
Week 12	0.2 (0.2–0.2)	0.2 (0.2–0.3)†††	0.2 (0.2–0.2)	0.2 (0.1–0.2)
Week 24	0.2 (0.1–0.3)	0.2 (0.2–0.2)	0.2 (0.2–0.2)	0.2 (0.2–0.3)¶¶
Week 36	0.2 (0.2–0.2)	0.2 (0.2–0.3)†††	0.2 (0.2–0.2)	0.2 (0.2–0.3)
Week 52	0.2 (0.2–0.2)	0.2 (0.2–0.2)	0.2 (0.2–0.2)	0.2 (0.1–0.3)
Week 68	0.2 (0.1–0.2)	0.2 (0.2–0.2)	0.2 (0.2–0.3)	0.2 (0.1–0.3)
**CH50, n (%)**				
Baseline, N	8	17	19	16
<10 U/mL	0 (0.0)	0 (0.0)	1 (5.3)	1 (6.3)
10–30 U/mL	1 (12.5)	0 (0.0)	0 (0.0)	0 (0.0)

>30–60 U/mL	1 (12.5)	7 (41.2)	7 (36.8)	9 (56.3)
>60 U/mL	6 (75.0)	10 (58.8)	11 (57.9)	6 (37.5)
Week 12, N	8	17	18	18
<10 U/mL	0 (0.0)	0 (0.0)	1 (5.6)	1 (6.3)
10–30 U/mL	0 (0.0)	0 (0.0)	0 (0.0)	0 (0.0)
>30–60 U/mL	2 (25.0)	6 (35.3)	7 (38.9)	8 (50.0)
>60 U/mL	6 (75.0)	11 (64.7)	10 (55.6)	7 (43.8)
Week 24, N	8	17	19	15
<10 U/mL	0 (0.0)	0 (0.0)	1 (5.3)	0 (0.0)
10–30 U/mL	0 (0.0)	0 (0.0)	0 (0.0)	1 (6.7)
>30–60 U/mL	2 (25.0)	5 (29.4)	5 (26.3)	3 (20.0)
>60 U/mL	6 (75.0)	12 (70.6)	13 (68.4)	11 (73.3)
Week 36, N	8	16	18	15
<10 U/mL	0 (0.0)	0 (0.0)	1 (5.6)	1 (6.7)
10–30 U/mL	0 (0.0)	0 (0.0)	0 (0.0)	1 (6.7)
>30–60 U/mL	1 (12.5)	3 (18.8)	6 (33.3)	3 (20.0)
>60 U/mL	7 (87.5)	13 (81.3)	11 (61.1)	10 (66.7)

Week 52, N	8	17	9	16
<10 U/mL	0 (0.0)	0 (0.0)	1 (5.3)	1 (6.3)
10–30 U/mL	0 (0.0)	0 (0.0)	0 (0.0)	1 (6.3)
>30–60 U/mL	3 (37.5)	5 (29.4)	4 (21.1)	4 (25.0)
>60 U/mL	5 (62.5)	12 (70.6)	14 (73.7)	10 (62.5)
Week 68, N	8	17	19	16
<10 U/mL	0 (0.0)	0 (0.0)	1 (5.3)	1 (6.3)
10–30 U/mL	0 (0.0)	0 (0.0)	0 (0.0)	1 (6.3)
>30–60 U/mL	2 (25.0)	4 (23.5)	4 (21.1)	4 (25.0)
>60 U/mL	6 (75.0)	13 (76.5)	14 (73.7)	10 (62.5)
**Serum kappa light chain protein, mean ± SD mg/L**				
Baseline	48.1 ± 29.6	30.5 ± 16.7	99.8 ± 241.5	40.6 ± 37.4
Week 12	39.8 ± 33.6	21.7 ± 12.3	95.8 ± 270.0	30.2 ± 24.0
Week 24	46.1 ± 30.0	20.4 ± 12.0	75.3 ± 145.7	27.1 ± 24.9[¶¶]
Week 36	50.7 ± 45.7	20.2 ± 11.8	84.0 ± 198.9***	25.7 ± 16.1
Week 52	55.8 ± 61.3	22.5 ± 13.3	61.5 ± 124.3	28.5 ± 21.6
Week 68	52.8 ± 45.3	23.1 ± 12.9	85.9 ± 180.0	28.8 ± 28.9

**Serum lambda light chain protein, mean ± SD mg/L**				
Baseline	41.9 ± 31.0	22.0 ± 7.3	23.5 ± 10.6	21.4 ± 9.4
Week 12	36.9 ± 28.6	18.7 ± 5.0	18.8 ± 6.7	18.9 ± 7.3
Week 24	42.0 ± 30.9	17.3 ± 4.6	19.2 ± 7.8	18.0 ± 6.1[¶¶]
Week 36	39.5 ± 35.1	16.7 ± 4.6	18.7 ± 6.9***	18.6 ± 6.0
Week 52	38.9 ± 40.0	16.9 ± 5.0	18.0 ± 5.9	19.5 ± 7.1
Week 68	39.9 ± 33.4	18.3 ± 6.4	19.1 ± 7.4	19.9 ± 9.5
**Kappa:lambda ratio, mean ± SD**				
Baseline	1.2 ± 0.5	1.3 ± 0.4	13.0 ± 48.2	1.7 ± 0.8
Week 12	1.0 ± 0.3	1.1 ± 0.4	23.0 ± 91.3	1.5 ± 0.6
Week 24	1.1 ± 0.2	1.1 ± 0.4	7.5 ± 20.2	1.4 ± 0.7[¶¶]
Week 36	1.2 ± 0.5	1.2 ± 0.4	9.1 ± 27.9***	1.3 ± 0.5
Week 52	1.4 ± 0.4	1.3 ± 0.5	5.5 ± 14.8	1.3 ± 0.6
Week 68	1.3 ± 0.4	1.2 ± 0.5	7.0 ± 16.7	1.3 ± 0.5
**Beta-2 microglobulin, mean ± SD nmol/L**				
Baseline	305.7 ± 159.9	226.8 ± 80.1	211.4 ± 45.5	239.0 ± 99.4

Week 12	329.5 ± 160.7	206.6 ± 61.7	213.0 ± 50.9	239.0 ± 98.4
Week 24	342.1 ± 154.7	216.0 ± 64.8	218.2 ± 57.5	242.4 ± 84.7[¶¶]
Week 36	342.1 ± 169.4	214.0 ± 66.6[†††]	211.8 ± 46.1	256.4 ± 96.0[¶¶]
Week 52	346.3 ± 146.3	221.3 ± 77.9	212.8 ± 36.0	254.0 ± 107.7
Week 68	375.2 ± 226.3	243.9 ± 92.1	230.2 ± 50.0	262.0 ± 130.0
**CXCL13, chemokine (C-X-C motif) ligand 13 (CXCL13), median (IQR) ng/L**				
Baseline	119.1 (82.9–183.5)	117.8 (89.3–244.9)	184.0 (102.6–406.4)	178.7 (112.1–272.5)
Week 12	149.5 (95.1–175.1)	54.9 (50.5–121.3)	134.5 (62.7–217.3)	154.3 (82.9–258.5)
Week 24	131.2 (109.9–212.9)	59.8 (34.7–131.5)	149.7 (95.2–184.9)	96.0 (63.3–182.1)[¶¶]
Week 36	165.8 (98.6–195.2)	80.4 (53.5–155.8)[†††]	123.8 (82.8–161.9)	138.0 (82.1–231.1)
Week 52	154.1 (88.0–231.1)	97.7 (59.8–158.6)	119.8 (70.1–251.1)	137.0 (92.1–288.0)
Week 68	114.3 (72.8–259.4)	134.2 (83.2–231.7)	148.6 (85.9–196.2)	147.0 (88.7–276.2)
**SS-A antibody, n (%)**				
Baseline, N	8	17	19	16
<7 KU/L	0 (0.0)	0 (0.0)	0 (0.0)	1 (6.0)
7–10 KU/L	0 (0.0)	0 (0.0)	0 (0.0)	0 (0.0)

>10–120 KU/L	0 (0.0)	1 (5.9)	0 (0.0)	3 (19.0)
>120–240 KU/L	0 (0.0)	0 (0.0)	2 (10.5)	2 (13.0)
>240 KU/L	8 (100.0)	16 (94.1)	17 (89.5)	10 (63.0)
Week 12, N	8	17	19	16
<7 KU/L	0 (0.0)	0 (0.0)	0 (0.0)	0 (0.0)
7–10 KU/L	0 (0.0)	0 (0.0)	0 (0.0)	0 (0.0)
>10–120 KU/L	0 (0.0)	1 (5.9)	1 (5.3)	3 (19.0)
>120–240 KU/L	0 (0.0)	2 (11.8)	1 (5.3)	1 (6.0)
>240 KU/L	8 (100.0)	14 (82.4)	17 (89.5)	12 (75.0)
Week 24, N	8	17	19	15
<7 KU/L	0 (0.0)	0 (0.0)	0 (0.0)	0 (0.0)
7–10 KU/L	0 (0.0)	0 (0.0)	0 (0.0)	0 (0.0)
>10–120 KU/L	0 (0.0)	2 (11.8)	1 (5.3)	4 (27.0)
>120–240 KU/L	0 (0.0)	0 (0.0)	0 (0.0)	0 (0.0)
>240 KU/L	8 (100.0)	15 (88.2)	18 (94.7)	11 (73.0)
Week 36, N	8	16	19	16
<7 KU/L	0 (0.0)	0 (0.0)	0 (0.0)	0 (0.0)

7–10 KU/L	0 (0.0)	0 (0.0)	0 (0.0)	0 (0.0)
>10–120 KU/L	0 (0.0)	1 (6.3)	1 (5.3)	4 (25.0)
>120–240 KU/L	0 (0.0)	1 (6.3)	3 (15.8)	0 (0.0)
>240 KU/L	8 (100.0)	14 (87.5)	15 (78.9)	12 (75.0)
Week 52, N	8	17	19	16
<7 KU/L	0 (0.0)	0 (0.0)	0 (0.0)	0 (0.0)
7–10 KU/L	0 (0.0)	0 (0.0)	0 (0.0)	1 (6.0)
>10–120 KU/L	0 (0.0)	1 (5.9)	1 (5.3)	3 (19.0)
>120–240 KU/L	0 (0.0)	1 (5.9)	1 (5.3)	0 (0.0)
>240 KU/L	8 (100.0)	15 (88.2)	17 (89.5)	12 (75.0)
Week 68, N	8	17	19	16
<7 KU/L	0 (0.0)	0 (0.0)	0 (0.0)	0 (0.0)
7–10 KU/L	0 (0.0)	0 (0.0)	0 (0.0)	2 (13.0)
>10–120 KU/L	0 (0.0)	2 (11.8)	1 (5.3)	2 (13.0)
>120–240 KU/L	0 (0.0)	0 (0.0)	2 (10.5)	0 (0.0)
>240 KU/L	8 (100.0)	15 (88.2)	16 (84.2)	12 (75.0)
**SS-B antibody, n (%)**				

Baseline, N	8	17	19	16
<10 KU/L	0 (0.0)	8 (47.1)	6 (31.6)	6 (37.5)
10–80 KU/L	2 (25.0)	4 (23.5)	3 (15.8)	4 (25.0)
>80–160 KU/L	1 (12.5)	1 (5.9)	1 (5.3)	1 (6.3)
>160–320 KU/L	3 (37.5)	3 (17.6)	2 (10.5)	0 (0.0)
>320 KU/L	2 (25.0)	1 (5.9)	7 (36.8)	5 (31.3)
Week 12, N	8	17	19	16
<10 KU/L	0 (0.0)	9 (52.9)	6 (31.6)	6 (37.5)
10–80 KU/L	2 (25.0)	3 (17.6)	3 (15.8)	4 (25.0)
>80–160 KU/L	2 (25.0)	2 (11.8)	1 (5.3)	1 (6.3)
>160–320 KU/L	2 (25.0)	3 (17.6)	2 (10.5)	0 (0.0)
>320 KU/L	2 (25.0)	0 (0.0)	7 (36.8)	5 (31.3)
Week 24, N	8	17	19	15
<10 KU/L	0 (0.0)	11 (64.7)	6 (31.6)	6 (40.0)
10–80 KU/L	2 (25.0)	2 (11.8)	3 (15.8)	5 (33.3)
>80–160 KU/L	2 (25.0)	1 (5.9)	1 (5.3)	0 (0.0)
>160–320 KU/L	2 (25.0)	3 (17.6)	1 (5.3)	0 (0.0)

>320 KU/L	2 (25.0)	0 (0.0)	8 (42.1)	4 (26.7)
Week 36, N	8	16	19	16
<10 KU/L	0 (0.0)	10 (62.5)	6 (31.6)	6 (37.5)
10–80 KU/L	2 (25.0)	3 (18.8)	3 (15.8)	4 (25.0)
>80–160 KU/L	3 (37.5)	1 (6.3)	1 (5.3)	1 (6.3)
>160–320 KU/L	1 (12.5)	2 (12.5)	1 (5.3)	0 (0.0)
>320 KU/L	2 (25.0)	0 (0.0)	8 (42.1)	5 (31.3)
Week 52, N	8	17	19	16
<10 KU/L	0 (0.0)	11 (64.7)	6 (31.6)	7 (43.8)
10–80 KU/L	3 (37.5)	2 (11.8)	3 (15.8)	3 (18.8)
>80–160 KU/L	2 (25.0)	1 (5.9)	1 (5.3)	1 (6.3)
>160–320 KU/L	1 (12.5)	3 (17.6)	1 (5.3)	0 (0.0)
>320 KU/L	2 (25.0)	0 (0.0)	8 (42.1)	5 (31.3)
Week 68, N	8	17	19	16
<10 KU/L	1 (12.5)	10 (58.8)	6 (31.6)	6 (37.5)
10–80 KU/L	2 (25.0)	3 (17.6)	3 (15.8)	4 (25.0)
>80–160 KU/L	1 (12.5)	1 (5.9)	1 (5.3)	1 (6.3)

>160–320 KU/L	2 (25.0)	3 (17.6)	1 (5.3)	0 (0.0)
>320 KU/L	2 (25.0)	0 (0.0)	8 (42.1)	5 (31.3)

*Of patients with positive RF values at baseline; [†]n=6; [‡]n=7; [§]n=14; [¶]n=11; **n=12; [††]n=9; [‡‡]n=5; [§§]n=10; [¶¶]n=15; ***n=18; [†††]n=16

BEL, belimumab; BLyS, B lymphocyte stimulator; CH50, hemolytic complement; Ig, immunoglobulin; IQR, interquartile range; PBO, placebo; RF, rheumatoid factor; RTX, rituximab; SD, standard deviation.

Supplementary Table 3. Minor salivary gland biomarkers over time (completer population, N=60)

MSG biopsy biomarker, median (IQR)	PBO (N=8)	BEL+RTX (N=17)	BEL (N=19)	RTX (N=16)
**Lymphocyte focus score, count per 4 mm^2**				
Baseline	3.3 (1.5–4.3)	2.1 (1.1–3.6)	2.5 (1.0–4.6)	2.1 (0.5–4.2)*
Week 24	2.1 (1.4–4.1)	1.6 (0.5–2.2)[†]	2.5 (1.4–6.9)[‡]	1.7 (0.8–3.6)*
**B cells (CD20+), count/mm^2**				
Baseline	418.2 (20.7–763.1)	87.5 (13.3–252.0)[‡]	65.9 (4.5–636.3)	106.3 (30.9–235.1)[§]
Week 24	93.4 (55.2–518.2)	0.4 (0.0–1.4)[¶]	60.6 (13.2–454.5)[‡]	158.3 (2.4–744.8)[†]
**T cells (CD3+), count/mm^2**				
Baseline	282.9 (122.8–656.4)	129.4 (75.4–333.3)[‡]	206.9 (53.8–715.8)	145.2 (47.3–351.6)*
Week 24	186.9 (34.6–470.8)	124.6 (39.9–178.9)[†]	255.4 (81.9–1080.1)[‡]	129.9 (26.4–244.3)*
**B cell (CD20+)/T cell (CD3+), ratio**				
Baseline	0.6 (0.3–0.7)	0.3 (0.2–0.9)[§]	0.2 (0.1–0.3)	0.6 (0.3–0.9)[†]
Week 24	0.4 (0.3–0.6)	0.0 (0.0–0.1)**	0.2 (0.1–0.7)*	0.4 (0.2–0.7)[¶]
**Plasma cells (CD138+; both CD20+ and CD20-), count/mm^2**				

Baseline	90.2 (5.7–742.9)	33.7 (7.2–198.0)††	117.6 (27.3–199.4)	35.8 (19.3–226.1)*
Week 24	188.0 (22.5–318.8)	101.6 (15.2–384.5)†	94.3 (17.0–204.6)‡	93.6 (17.5–248.6)*
**Total aggregate area/total glandular area, ratio**				
Baseline	0.088 (0.023–0.144)	0.021 (0.010–0.045)	0.046 (0.009–0.140)	0.042 (0.004–0.090)*
Week 24	0.032 (0.019–0.111)	0.009 (0.006–0.020)†	0.024 (0.013–0.200)‡	0.033 (0.017–0.066)*
**Average focus size, mm^2**				
Baseline	0.092 (0.053–0.120)	0.043 (0.026–0.062)	0.058 (0.024–0.145)	0.064 (0.015–0.103)*
Week 24	0.081 (0.035–0.128)	0.032 (0.020–0.047)†	0.038 (0.022–0.135)‡	0.053 (0.040–0.128)*
**Foci displaying germinal centres, %**				
Baseline	50.1 (12.5–63.5)	5.6 (0.0–29.9)††	20.5 (0.0–46.4)‡‡	23.6 (0.0–39.3)§
Week 24	4.2 (0.0–45.5)	0.0 (0.0–18.2)§§	25.0 (0.0–45.2)*	23.8 (7.1–66.7)§
**Foci displaying follicular dendritic cells, %**				
Baseline	48.5 (12.5–70.5)	16.7 (6.3–26.9)††	45.8 (0.0–50.0)‡‡	33.3 (25.0–51.8)§
Week 24	15.6 (0.0–51.8)	0.0 (0.0–23.5)§§	25.0 (0.0–40.7)*	36.5 (22.5–66.7)§
**Foci displaying CD3/CD20 segregation, %**				
Baseline	35.7 (6.3–48.2)	5.0 (0.0–15.2)††	16.7 (8.3–50.0)‡‡	27.5 (0.0–33.0)§
Week 24	4.2 (0.0–37.0)	0.0 (0.0–2.4)§§	6.7 (0.0–50.0)*	16.7 (0.0–33.3)§

**Memory B cells (CD20+ CD27+),¶¶ count/mm^2**				
Baseline	114.9 (18.3–388.8)***	14.4 (3.9–41.3)††	31.0 (1.4, 141.1)	95.3 (1.9, 219.7)§§
Week 24	57.0 (16.0–419.6)***	0.8 (0.0, 7.7)¶	57.0 (4.4, 437.0)‡	2.6 (0.2, 66.8)§§
**Non-switched memory B cells (IgD+ CD20+ CD27+),¶¶ count/mm^2**				
Baseline	1.2 (0.0–1.5)***	0.0 (0.0–0.2)††	0.0 (0.0–0.5)	0.9 (0.1–4.2)§§
Week 24	1.1 (0.1–5.9)***	0.1 (0.0–7.5)¶	0.1 (0.0–2.9)‡	0.0 (0.0–0.2)§§
**Switched memory B cells (IgD- CD20+ CD27+),¶¶ count/mm^2**				
Baseline	114.9 (17.0–387.3)***	14.3 (3.9–40.2)††	31.0 (1.4–140.9)	91.2 (1.8–215.5)§§
Week 24	54.1 (15.9–353.8)***	0.1 (0.01.3)¶	57.0 (4.4–436.2)‡	0.5 (0.0–66.8)§§
**Follicular B cells (IgD+ CD20+), count/mm^2**				
Baseline	0.1 (0.0–1.6)***	0.0 (0.0–0.6)††	0.0 (0.0–0.6)	0.1 (0.0–1.6)§§
Week 24	0.3 (0.0–1.1)***	0.0 (0.0–0.0)¶	0.0 (0.0–0.5)‡	0.0 (0.0–0.5)§§

*n=14; †n=12; ‡n=15; §n=13; ¶n=10; **n=8; ††n=16; ‡‡n=17; §§n=11; ¶¶Due to technical difficulties with the CD27 stain in one patient, only 11 patients were assessed for memory B cells in the rituximab group; ***n=7

BEL, belimumab; Ig, immunoglobulin; IQR, interquartile range; MSG, minor salivary gland; PBO, placebo; RTX, rituximab.

Supplementary Table 4. Additional clinical outcome measures

**Variable – Completer population (N=60)**	**PBO (N=8)**	**BEL+RTX (N=17)**	**BEL (N=19)**	**RTX (N=16)**
ClinESSDAI responders*, n (%)				
Week 12	3 (37.5)	7 (41.2)	10 (52.6)	7 (43.8)
Week 24	3 (37.5)	9 (52.9)	7 (36.8)	7 (43.8)
Week 36	3 (37.5)	8 (47.1)	12 (63.2)	5 (31.3)
Week 52	3 (37.5)	10 (58.8)	11 (57.9)	6 (37.5)
Week 68	3 (37.5)	9 (52.9)	12 (63.2)	6 (37.5)
**Variable – Safety population (N=86)**	**PBO (N=13)**[†]	**BEL+RTX (N=24)**[†]	**BEL (N=24)**[†]	**RTX (N=25)**[†]
Lacrimal gland function (Schirmer's test, mm/min), mean ± SD				
Left eye				
Baseline	0.65 ± 0.837	1.03 ± 1.157	0.53 ± 0.598	0.98 ± 1.365
Week 12	0.40 ± 0.323	0.60 ± 1.394	0.40 ± 1.363	0.80 ± 1.460
Week 24	0.65 ± 0.830	0.82 ± 1.112	0.54 ± 0.796	0.86 ± 0.945
Week 52	0.56 ± 0.765	0.56 ± 0.654	0.70 ± 1.404	0.93 ± 1.530

Right eye				
Baseline	0.37 ± 0.345	1.01 ± 1.235	0.63 ± 0.783	0.66 ± 0.842
Week 12	0.43 ± 0.466	0.92 ± 1.458	1.08 ± 1.813	1.04 ± 1.123
Week 24	0.76 ± 1.379	0.52 ± 0.679	0.66 ± 1.232	0.70 ± 0.922
Week 52	0.66 ± 1.240	0.65 ± 1.097	0.77 ± 0.984	0.77 ± 1.012

*Responder defined as patients with a total ClinESSDAI score <5; †Numbers at baseline; numbers decreased over the study visits.

BEL, belimumab; ClinESSDAI, Clinical European League Against Rheumatism Sjögren's Syndrome Disease Activity Index; PBO, placebo; RTX, rituximab; SD, standard deviation.

Supplementary Table 5. Endpoints

Endpoints as defined for study	Population	Detail of measurements	Outcome as defined for paper
**Primary**	**Safety**		
Safety		Incidence of AEs, AESIs (malignant neoplasms, PASR, all infections of special interest [opportunistic infections, herpes zoster, tuberculosis, sepsis], depression/suicide/self-injury), study-specific AESIs (severe skin reactions, cardiac disorders, posterior reversible encephalopathy syndrome, progressive multifocal leukocephalopathy, and biopsy-related AEs) and deaths until week 68	Safety outcome
**Secondary**	**Safety and completer (presented for completer)**		
MSG CD20+ B cells		Change in absolute count/mm^2 at baseline versus and week 24	Immunological outcome

ESSDAI		Mean ESSDAI total score over time to week 68, the proportion of ESSDAI responders to week 68 (category 1: ≥3-point improvement in total ESSDAI versus baseline; category 2: ≥5-point improvement in total ESSDAI versus baseline; category 3: ESSDAI total score <5)	Clinical outcome
Stimulated salivary flow		Mean stimulated salivary flow over time to week 68	Clinical outcome
Oral dryness		NRS to week 68	Clinical outcome
**Key exploratory and health outcome**	**Safety and completer (presented for completer)**		
B cells (total [CD19+], memory [CD20+ CD27+], naïve [CD20+ CD27-] and plasmablast [CD27br+ CD38br+ CD19+])		Number of B cells measured by flow cytometry to week 68	Immunological outcome
MSG biomarkers (LFS, B cells, B-cell/T-cell ratio, plasma cells, total		Change in MSG biomarkers over time to week 68	Immunological outcome

aggregate area/total glandular area ratio, average focus size, foci displaying germinal centers, foci displaying follicular dendritic cells, foci displaying CD3/CD20 segregation, plasma cell/B-cell ratio, memory B cells [switched and non-switched], follicular B cells)			
Histological assessment of MSG biopsy		Histological assessments of salivary gland biopsy samples at baseline versus week 24	Immunological outcome
Serological biomarkers (IgG, RF, IgA, IgM, free BLyS, total BLyS, C3, C4, CH50, kappa and lambda light chain, kappa:lambda ratio, beta2 microglobulin, CXCL13, SS-A, SS-B)		Change in biomarkers over time	Immunological outcome
ESSPRI		Mean ESSPRI over time to week 68, by total score and domain (dryness, fatigue, and pain)	Clinical outcome

Lacrimal gland function (Schirmer's test)		Lacrimal gland function over time	Clinical outcome
Unstimulated salivary flow		Changes from baseline in unstimulated salivary flow	Clinical outcome

AE, adverse event; AESI, adverse event of special interest; br, bright; C3/C4, component 3/4; CXCL13, chemokine (C-X-C motif) ligand 13; ESSDAI, EULAR Sjögren's Syndrome Disease Activity Index; ESSPRI, EULAR Sjögren's Syndrome Patient Reported Index; EULAR, European League Against Rheumatism; Ig, immunoglobulin; LFS, lymphocyte focus score; MSG, minor salivary gland; NRS, Numeric Response/Rating Scale; PASR, post-administration systemic reaction; RF, rheumatoid factor.
